# Deficiency of Mineralization-Regulating Transcription Factor Trps1 Compromises Quality of Dental Tissues and Increases Susceptibility to Dental Caries

**DOI:** 10.3389/fdmed.2022.875987

**Published:** 2022-04-11

**Authors:** Mairobys Socorro, Priyanka Hoskere, Catherine Roberts, Lyudmila Lukashova, Kostas Verdelis, Elia Beniash, Dobrawa Napierala

**Affiliations:** 1 Center for Craniofacial Regeneration, Department of Oral and Craniofacial Sciences, University of Pittsburgh School of Dental Medicine, Pittsburgh, PA, United States,; 2 Department of Restorative Dentistry/Comprehensive Care, University of Pittsburgh, School of Dental Medicine, Pittsburgh, PA, United States,; 3 Department of Endodontics and Center for Craniofacial Regeneration, University of Pittsburgh, School of Dental Medicine, Pittsburgh, PA, United States,; 4 McGowan Institute for Regenerative Medicine, University of Pittsburgh, Pittsburgh, PA, United States

**Keywords:** odontoblasts, mineralization, conditional knockout mice, dental caries, trichorhinophalangeal syndrome

## Abstract

Dental caries is the most common chronic disease in children and adults worldwide. The complex etiology of dental caries includes environmental factors as well as host genetics, which together contribute to inter-individual variation in susceptibility. The goal of this study was to provide insights into the molecular pathology underlying increased predisposition to dental caries in trichorhinophalangeal syndrome (TRPS). This rare inherited skeletal dysplasia is caused by mutations in the *TRPS1* gene coding for the TRPS1 transcription factor. Considering *Trps1* expression in odontoblasts, where Trps1 supports expression of multiple mineralization-related genes, we focused on determining the consequences of odontoblast-specific *Trps1* deficiency on the quality of dental tissues. We generated a conditional *Trps1*^*Col1a1*^ knockout mouse, in which *Trps1* is deleted in differentiated odontoblasts using *2.3kbCol1a1-Cre*^*ERT*2^ driver. Mandibular first molars of 4wk old male and female mice were analyzed by micro-computed tomography (μCT) and histology. Mechanical properties of dentin and enamel were analyzed by Vickers microhardness test. The susceptibility to acid demineralization was compared between WT and *Trps1*^*Col1a1*^cKO molars using an *ex vivo* artificial caries procedure. μCT analyses demonstrated that odontoblast-specific deletion of *Trps1* results in decreased dentin volume in male and female mice, while no significant differences were detected in dentin mineral density. However, histology revealed a wider predentin layer and the presence of globular dentin, which are indicative of disturbed mineralization. The secondary effect on enamel was also detected, with both dentin and enamel of *Trps1*^*Col1a1*^cKO mice being more susceptible to demineralization than WT tissues. The quality of dental tissues was particularly impaired in molar pits, which are sites highly susceptible to dental caries in human teeth. Interestingly, *Trps1*^*Col1a1*^cKO males demonstrated a stronger phenotype than females, which calls for attention to genetically-driven sex differences in predisposition to dental caries. In conclusion, the analyses of *Trps1*^*Col1a1*^cKO mice suggest that compromised quality of dental tissues contributes to the high prevalence of dental caries in TRPS patients. Furthermore, our results suggest that TRPS patients will benefit particularly from improved dental caries prevention strategies tailored for individuals genetically predisposed due to developmental defects in tooth mineralization.

## INTRODUCTION

Dental caries remains the most prevalent chronic disease affecting 60–90% of children and adolescents (5–17 years of age), and nearly every adult among all populations (1–4). This complex multifactorial disease involves progressive destruction or demineralization of teeth, caused by acids produced by commensal bacteria within oral biofilms (5–7). Initially, dental caries affects only enamel. As the caries lesion progresses, crown dentin becomes affected. Cementum and root dentin are only affected when roots of teeth are exposed to the oral cavity, which occurs mostly in elderly patients (8). Of note, individual tooth surfaces have vastly different susceptibilities to caries, with the occlusal pits and fissures of molars being the most susceptible (9, 10).

Many environmental, endogenous, and behavioral risk factors have been identified as contributors to the development of dental caries (4, 11, 12). Nevertheless, evidence from genome-wide association studies (GWAS), complex segregation analysis and twin as well as family-based studies indicate that susceptibility to dental caries is strongly influenced by each individual’s genetic constitution, which dictates tooth anatomy (e.g., the depth of pits and fissures), quality of dental mineralized tissues, salivary properties, host immunity and taste preference (13–18). One potential mechanism of a genetic contribution to dental caries is the formation of hypomineralized dental tissues that are more prone to acid-caused destruction (19). This is, for example, the case in genetic diseases such as dentinogenesis imperfecta and hypophosphatasia, providing evidence that high susceptibility to dental caries initiation and progression is associated with decreased tooth mineral content (19, 20).

Mineralization of dental tissues depends on multiple factors, including protein composition of the extracellular matrix (ECM), post-translational modifications of ECM proteins, availability of calcium (Ca^2+^) and phosphate (P_i_) ions, pH, and the presence of mineralization inhibitors (21–23). Additionally, proper transcriptional regulation of the function of cells producing mineralized ECM is crucial for formation of sound mineralized tissues (24–26). This has been well demonstrated for two major mineralization-supporting transcription factors: Runx2 and Osx/Sp7. Specifically, a significant reduction in mineralization and size of enamel rods was reported in *Runx2* conditional knockout (cKO) mice (26), while studies of *Osx/Sp7* null mice revealed disrupted differentiation of both odontoblasts and ameloblasts, affecting tooth mineralization (25). Although, these *in vivo* studies highlighted the importance of transcriptional regulation in the formation of dental tissues, the contribution of mineralization-regulating transcription factors in the predisposition to dental caries has yet to be elucidated, in spite of evidence from GWAS studies identifying genes coding for transcription factors as dental caries risk loci (27, 28).

Trps1 is a transcription factor involved in tooth development and mineralization of skeletal and dental tissues (29–34). Trps1 is encoded by the *Trps1* gene located on the distal half of the chromosome 15 in the mouse genome, and on the long arm of the chromosome 8, at 8q24, in the human genome (31). The name of this protein and the gene come from the trichorhinophalangeal syndrome (TRPS), a rare autosomal-dominant skeletal dysplasia caused by mutations in the *TRPS1* gene (31). Moreover, the name of this condition refers to some of the most characteristic features observed in affected individuals: tricho—derives from the Greek “trikhos”, meaning “hair”; rhino-comes from the Greek “rhís”, meaning “nose”; while phalangeal-refers collectively to the digital bones in the hands and feet. Patients with this syndrome have fine, sparse, and slow-growing scalp hair, a characteristic bulbous nose with a broad nasal septum and tip, and short digits with radiographically visible cone-shaped epiphyses restricted to the middle or proximal phalangeal bones (33, 35). In addition to epiphyseal defects, characteristic to TRPS is short stature, hip dysplasia and premature closure of growth plates (31, 35), all indicative of defective development of endochondral bones. TRPS patients also present with hypoplastic mandibular condyles, reduced bone mineral density (osteopenia), and midface hypoplasia (32, 36, 37). Severe osteoporosis has been reported in some affected individuals (38).

The array and severity of phenotypic manifestations of TRPS differ among affected individuals and even within family members (33, 35). There is, however, some genotype-phenotype correlation, which allowed to distinguish three types of TRPS. TRPS type I (OMIM#190350) is a milder form, caused by nonsense mutations resulting in *TRPS1* haploinsufficiency. More severe TRPS type III (OMIM#190351) is caused by missense mutations located exclusively in the exon 6 and 7 of *TRPS1* gene, resulting in functional modification of the DNA binding domain of the TRPS1 transcription factor (35, 39). These mutations are predicted to have a combination of a loss- and gain-of-function effect. TRPS type II or Langer-Giedion syndrome (OMIM#150230) is caused by a contiguous gene deletion, which, in addition to *TRPS1*, also encompasses the adjacent gene for multiple hereditary exostoses *EXT1* (40, 41). Hence, the phenotype of type II combines features of TRPS type I with multiple exostosis, while individuals with TRPS type III demonstrate severe brachydactyly and short stature (42, 43).

TRPS is one of a few genetic syndromes with frequently reported dental abnormalities, although most of the reports only briefly mention dental phenotypes, without their detailed characterization. Nonetheless, TRPS oral findings reported in (sparse) literature encompass defects in tooth number, size, shape, and mineralization. The most common TRPS dental abnormalities include supernumerarity, microdontia, malocclusion, and delay in root and crown development. Cases of hypodontia, abnormal tooth morphogenesis, impaired dentin mineralization, large dental pulp chambers, and extensive dental caries have also been reported, underscoring the clinical importance of *TRPS1* for tooth development (32, 33, 36, 44, 45).

Analyses of *Trps1* expression during mouse tooth organ development, demonstrated that *Trps1* is highly and specifically expressed in the dental mesenchyme (29). After cytodifferentiation, high *Trps1* expression becomes restricted to dental follicle cells, preodontoblasts and odontoblasts. The onset of dentin mineralization coincides with transient downregulation of *Trps1* expression in odontoblasts (29, 46). This dynamic expression pattern suggests that *Trps1* is involved in odontoblast differentiation and function, hence in dentin formation, suggesting that the susceptibility to dental caries reported in TRPS patients might result from formation of compromised dental mineralized tissues. This is further supported by our previous *in vitro* studies of 17IIA11 odontoblast cells showing that *Trps1* deficiency results in loss of their ability to initiate mineralization (47). This was accompanied by significant downregulation of the key osteogenic transcription factor Osx/Sp7 and other mineralization-related genes in *Trps1*-deficient 17IIA11 cells in comparison with control cells. These *in vitro* studies suggested that Trps1 is required for odontoblast-driven mineralization.

Two different genetic mouse models were independently generated to study Trps1 functions in development and mechanisms underlying TRPS pathologies. The first one (*Trps1*^Δ*gt*^ mice), harbors an allele with the in-frame deletion of the exon coding for the GATA-type DNA binding domain of Trps1 (48). The second one harbors a true null allele generated by deleting the first coding exon of *Trps1* (49). *Trps1*^−/−^ and *Trps1*^Δ*gt*/Δ*gt*^ mice have similar, although not identical, phenotypes. Both models demonstrate delayed cartilage development and endochondral bone formation (34, 48–50), craniofacial skeleton abnormalities (48, 49, 51), and reduced number of hair follicles (48, 49). No apparent tooth developmental abnormalities were observed in *Trps1*^Δ*gt*/Δ*gt*^ newborn mice (29), while mild decrease of mineralization in dental tissues was detected by micro-computed tomography (μCT) in 4wk old *Trps1*^+/Δ*gt*^ mice (30). However, due to neonatal lethality in the homozygous form and very mild phenotypes of heterozygous mice, these models are not suitable for studies addressing consequences of *Trps1* deficiency on the postnatal development. This particularly hampers studies of the tooth, since in mice, development of dental tissues and their mineralization occurs mostly postnatally (52).

This project builds upon the clinical findings of high prevalence of dental caries in TRPS patients, as well as data from *in vitro* studies of *Trps1*-deficient odontoblast cell line and animal models, which collectively suggest an important role of Trps1 in formation of dentin. We hypothesize that deficiency of *Trps1* in odontoblasts results in impaired quality of dentin and causes a secondary effect on enamel, which makes dental tissues more prone to acid-caused destruction. To investigate the role of the Trps1 transcription factor in the quality of dental mineralized tissues, we generated a cKO mouse with targeted deletion of the *Trps1* gene in odontoblasts. This new animal model overcomes the limitations of the conventional *Trps1* KO, which do not survive after birth, allowing to study the role of Trps1 in odontoblasts and postnatal development of dentin.

## MATERIALS AND METHODS

### Animals

**FIGURE 1 |:**
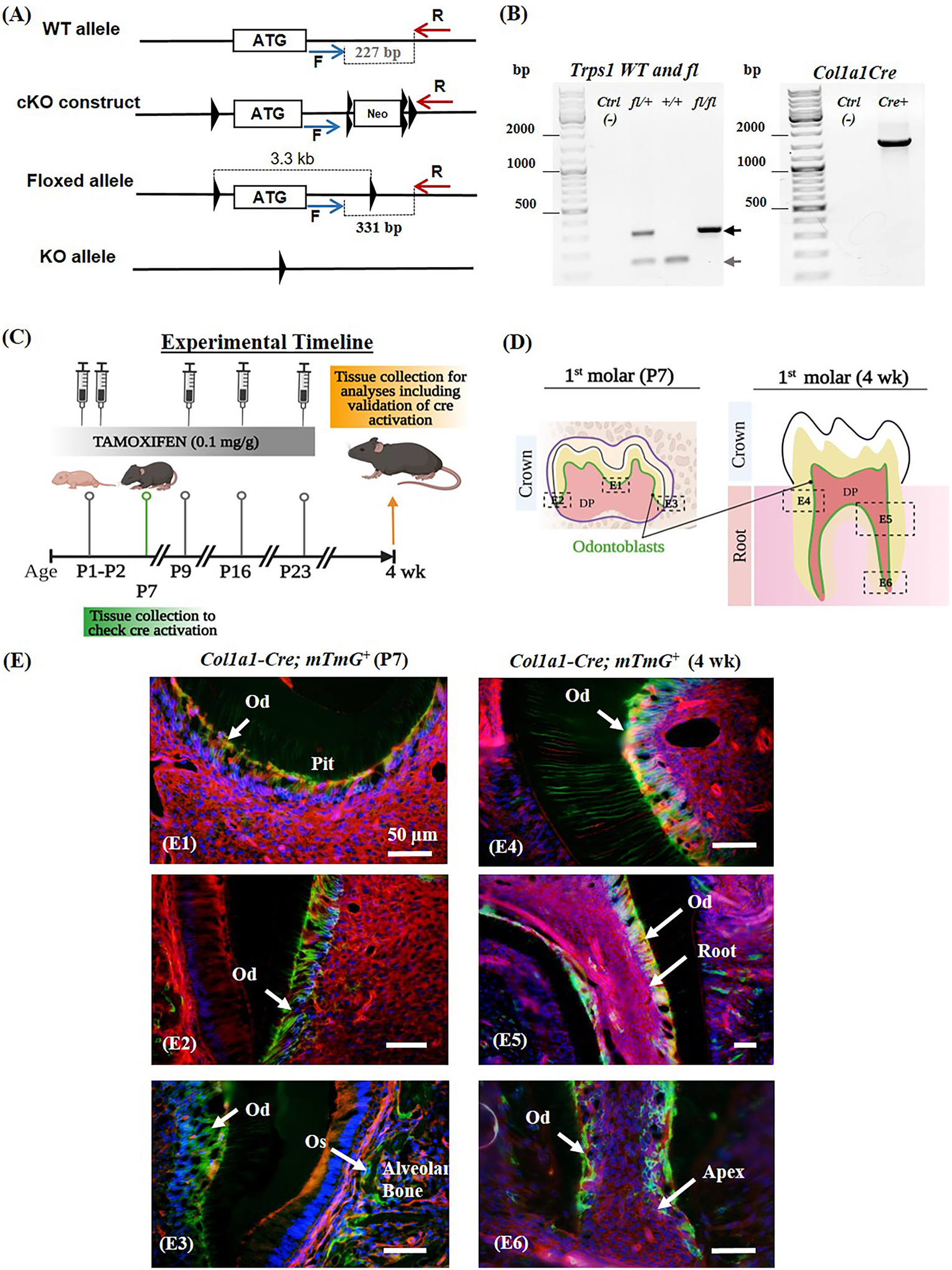
Generation of *Trps1*^*Col1a1*^cKO mice and verification of Cre-mediated recombination in odontoblasts. **(A)** The conditional knockout strategy: schematics of *Trps1* WT allele, targeting construct, and floxed allele showing the first coding exon (the white box with the ATG) and relative localization of LoxP sites (triangles), FRT sites (double triangles) and primers for genotyping PCRs (blue and red arrows). **(B)** Examples of genotyping PCR results: Floxed (*fl*; 331 bp) and WT (227 bp) alleles were detected by PCR with F and R primers, *Col1a1-Cre* transgene was detected with primers encompassing *Col1a1* promoter fragment and Cre sequence (57). **(C)** Experimental timeline showing the age of mice during tamoxifen administration, timing and dose of tamoxifen injections, and tissue collections. **(D)** Schematics of P7 and 4 wk old mouse molars showing the areas analyzed in panel E (dotted box); localization of cells of interest (odontoblasts) is shown in green. **(E)** Validation of the experimental timeline of tamoxifen injections and Cre-mediated recombination in odontoblasts using ROSA^*mTmG*^ reporter mice. Fluorescent microscopy images showing efficient and specific tamoxifen-induced Cre-mediated recombination (GFP+ signal; green and yellow cells) in odontoblasts (Od) and osteoblasts (Os) of mandibular first molars and alveolar bone, respectively. Red fluorescent cells are recombination negative. DAPI was used to stain nuclei (blue). (E1) Occlusal pit of P7 mouse molar, (E2) P7 crown dentin, (E3) P7 crown dentin and alveolar bone, (E4) 4 wk crown dentin, (E5) 4 wk molar root, (E6) 4 wk molar root apex. Scale bar = 50μm.

Male and female WT and *2.3kbCol1a1-Cre*^*ERT*2^*;Trps1*^*fl*/*fl*^ conditional knockout (*Trps1*^*Col1a1*^cKO) mice were used. To generate *Trps1* cKO mice, *Trps1* cKO (*Trps1*^*fl*^) allele was generated by inserting two LoxP sites flanking the first coding exon of *Trps1* by homologous recombination (Figure 1A). The deletion of this exon was used by Suemoto et al. (49), as a strategy to successfully generate the *Trps1* null allele. The *Trps1* cKO construct contained a neomycin resistance cassette (Neo^r^) flanked by FRT sites for a positive selection of recombinant embryonic stem cells (ES). Following the *Trps1* cKO construct injection into C57BL/6 ES cells, neomycin selection was performed, and the resistant ES clones were screened to verify the recombination. Mice carrying the recombinant allele were subsequently obtained *via* the generation of germline chimeras. The *Trps1*^*fl*^ allele was generated after breeding with germline deleter Flp mice (The Jackson Laboratory, strain # 003946)(53), which removed the Neo^r^ cassette. *Trps1*^*Col1a1*^cKO mice were generated by breeding *Trps1*^*fl*^ mice with *2.3kbCol1a1-Cre*^*ERT*2^ mice [B6.Cg-Tg (Col1a1-Cre/ERT2)1Crm/J, The Jackson Laboratory, strain # 016241] expressing Cre recombinase under the control of the 2.3-kb fragment of *Col1a1* promoter (Supplementary Figure 1) (54). The ERT2 domain renders Cre inactive in the cytoplasm, and tamoxifen releases Cre from the ERT2 inhibition allowing its translocation to the nucleus and recombination activity (55, 56). To activate Cre recombinase, tamoxifen (Sigma-Aldrich, # T5648) was administered to all experimental 2*.3kbCol1a1-Cre*^*ERT*2^*;Trps1*^*fl*/*fl*^ and control WT (*Trps1*^*fl*/*fl*^) mice *via* intraperitoneal injection (0.1 mg/g body weight) at postnatal days (P)1, P2, P9, P16 and P23 to assure efficient deletion of *Trps1* in odontoblasts and as a control in WT mice (Figure 1C). Tamoxifen was dissolved in corn oil to a concentration of 10 mg/ml. mTmG [B6.129(Cg)-Gt(ROSA)26Sortm4(ACTB-tdTomato,-EGFP)Luo/J, The Jackson Laboratory, strain # 007676] crossed with *2.3kbCol1a1-Cre*^*ERT*2^ mice were used to verify Cre-mediated recombination by microscopic imaging. Genotyping was carried out on the DNA extracted from tail biopsies using PCR with the following primers: WT and *Trps1*^*fl*^ allele (F: CCCATAGCACTTATTTAGTCCAG; R: CCTATCCTTTGTAACCTAACTCTC), Col1a1-Cre (F: CTCAGAGCTGTTATTTATTA; R: CATCGACCGGTAATGCAG) (57) (Figure 1B). The mice were kept on a caries-susceptible C57BL/6J genetic background (58).

All animal studies were conducted in accordance with a protocol approved by the University of Pittsburgh Institutional Animal Care and Use Committee (IACUC protocol # 19095648), complying with the Federal Animal Welfare Act and all NIH policies regarding vertebrate animals in research. Mice were euthanized by CO_2_ inhalation. All analyses were performed on tissues collected post-mortem.

### Histology

Hemimandibles of 4 wk old WT and *Trps1*^*Col1a1*^cKO male and female mice (*N* = 3/genotype/sex) were dissected under a stereo microscope (Leica S9D) to remove soft tissues, and fixed with 10% formalin (Fisher, # S7100–4) overnight. Samples were decalcified in 10% ethylenediaminetetraacetic acid (EDTA) solution (pH 7.4) for 14 days prior to paraffin embedding. Serial 7 μm sagittal sections were placed on Fisherbrand^™^ Superfrost^™^ Plus microscope slides and deparaffinized for hematoxylin and eosin (H&E) staining following standard protocols. Hemimandibles of P7 and 4 wk old *2.3kbCol1a1-Cre*^*ERT*2^;*mTmG* reporter mice were harvested and processed as described above. Decalcified samples were cryoprotected in 30% sucrose/PBS overnight at 4 °C, embedded in an OCT compound and stored at−80°C until sectioned. Samples were cryosectioned at 7 μm, protected from the light. To validate Cre recombinase activation in odontoblasts of mandibular first molars of reporter mice, cryosections were counterstained with DAPI and mounted with immu-mount (Fisher, # 9990402) for microscopic analyses of GFP and RFP signals.

### Microscopy

Whole teeth images were captured on a Leica M165FC dissecting microscope using a DFC 450 camera and Leica LAS software. Histological images were captured on a Zeiss AXIO microscope with an AxioCam MRc 35 camera and Zen software. Microindentation images were captured using a BUEHLER^®^ IndentaMet^™^1100 Series microindentation hardness tester adapted to a uEye camera and a Buehler Omnimet MHT software.

### μCT Scanning and Analyses

For the densitometric and volumetric analysis of mineralized tissues of mandibular first molars, hemimandibles of 4wk old mice (*N* = 5/genotype/sex) were imaged in 70% ethanol by the Scanco μCT 50 (Scanco Medical, Brüttisellen, Switzerland) system. The following parameters were set for the scans: 6-μm voxel size, 55 KVp, 0.36 degrees rotation step (180 degrees angular range) and a 1,500 ms exposure per view. After 3D reconstruction, volumes were segmented using a global threshold of 0.6 g HA/cc. Mineral density (TMD), thickness (Th), and volumes (BV) were measured for enamel and dentin separately. Additionally, dentin and enamel tissue fraction (BV/TV) in the total tooth crown volume (TV) was calculated as described before (59).

### Dental Tissues Microhardness Tests

Each first molar was extracted from the hemimandible after μCT scanning, mounted in Epofix (EMS, Hatfield, PA) and polished as previously described (60). Mechanical hardness was measured by doing three indentations with a Vickers diamond (25 g load during 5 s dwell time) using a microhardness tester (IndentaMet 1,100 Series, Buehler Ltd., Lake Bluff, IL, USA), on polished enamel (inner and outer) and dentin (mantle and circumpulpal) in the occlusal, middle, and cervical third of the crown. All indentations were made perpendicularly to the external surface of each specimen (61). Additionally, indentations in the enamel and dentin localized in the pit were made at a distance of at least three indentations diagonal from each other. The resulting indentations were measured under a microscope. Vickers hardness values were calculated using the following formula: HV = 1.854 × F/d^2^. With F being the applied load (measured in kg-force) and d^2^ the area of the indentation (measured in mm^2^) (62). Microhardness of each specific region of the enamel and dentin was expressed as a mean value of N=3 indentations.

### Artificial Caries Procedure

Molars of 4wk old WT and *Trps1*^*Col1a1*^cKO mice (N=5/genotype/sex) were analyzed using the protocol described by Vieira et al., (61). This method produces a subsurface enamel lesion instead of surface erosion (61). Briefly, following baseline microhardness measurements of enamel and crown dentin, artificial caries lesions were created by immersing each mounted molar in demineralizing solution (1.3 mmol/L Ca, 0.03 μg F/mL, 0.05 mol/L acetate buffer, phosphoric acid to adjust pH 5.0) at 37°C for 16 h. The post-demineralization indentations were created right underneath the baseline indentations on proximal surfaces, or next to the initial one in molar pits at a distance of at least one indentation diagonal from each other. We analyzed surface microhardness of inner and outer enamel, mantle and circumpulpal dentin, in addition to enamel and crown dentin in pits. The susceptibility to dental caries was expressed as a difference between mean values of pre- and post-demineralization mechanical hardness.

### Statistical Analyses

Experiments performed in this study used five mice per genotype per sex or otherwise stated. Males and females were analyzed separately. Values are expressed as mean ± standard deviation (SD). Statistically significant differences were determined using the Student’s *t*-test. A *p*-value of < 0.05 was considered statistically significant. Statistical analyses were performed using GraphPad Prism 9 software (GraphPad Software, La Jolla, CA, USA).

## RESULTS

### Generation of *2.3kbCol1a1-Cre*^*ERT*2^*;Trps1*^*fl*/*fl*^ Conditional Knockout (*Trps1*^*Col1a1*^cKO) Mice

Neonatal lethality of the current mouse models of *Trps1* deficiency (48, 49), limits *in vivo* studies of Trps1 function to embryonic development. To enable the research addressing the role of Trps1 in postnatal development and in specific cell types, we generated *Trps1* cKO mice, in which the first coding exon of *Trps1* is flanked by LoxP sites (*Trps1*^*fl*^ mice; Figure 1A). To determine consequences of *Trps1* deficiency on quality of dental tissues, which are formed postnatally in mice, and gain insights into the mechanism underlying increased susceptibility to dental caries in TRPS, we generated mice with deficiency of *Trps1* in odontoblasts (*Trps1*^*Col1a1*^cKO mice). *Trps1* knockout was initiated with tamoxifen injections at postnatal day 1 (P1) and P2, followed by 3 more injections 7 days apart (Figure 1C) (63–65). To verify the efficiency of this tamoxifen administration scheme in activating Cre recombination in odontoblasts, we used *2.3kbCol1a1*-Cre^ERT2^;*mTmG* reporter mice. Cre recombination was evaluated in molars of P7 and 4wk old reporter mice (Figures 1D,E). Fluorescent microscopy analyses detected cell membrane-localized GFP+ (green and yellow) signals in the odontoblast layer and RFP+ (red) signals in the remaining pulp cells. GFP+ signals were also detected in osteoblasts lining the alveolar bone, which is another cell type expressing Cre from the *2.3kbCol1a1* promoter (54). These results demonstrate that our tamoxifen administration scheme efficiently induced Cre recombination in odontoblasts as well as in osteoblasts in the alveolar bone (Figures 1D,E).

### Gross Characterization of *Trps1*^*Col1a1*^cKO Mice

**FIGURE 2 |:**
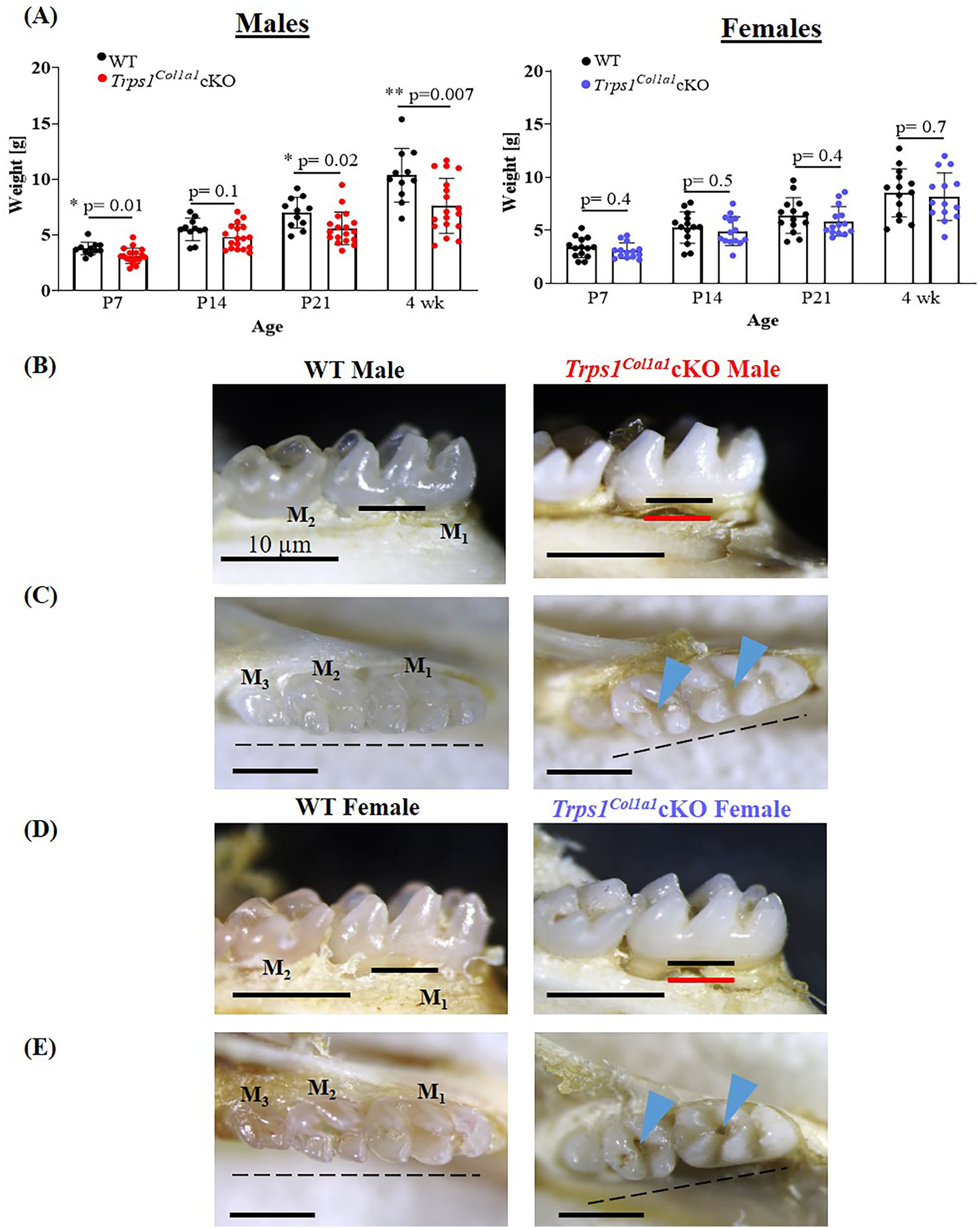
Gross assessment of *Trps1*^*Col1a1*^cKO mice and appearance of teeth. **(A)** Body weight of WT and *Trps1*^*Col1a1*^cKO mice (males, *N* = 11 and *N* = 18, respectively; females, *N* = 14 and *N* = 14, respectively) showing mild growth retardation of *Trps1*^*Col1a1*^cKO males. Data are shown as individual weight of each mouse (represented by dots) along with mean values ± SD; *p < 0.05 and **p < 0.01. **(B,D)** Lingual view of mandibular molars (M_1_ and M_2_) showing “chalky” white appearance and exposed root surfaces in 4 wk old *Trps1*^*Col1a1*^cKO male and female mice. The black line represents the bone level at baseline, and the red line represents the bone level in the *Trps1*^*Col1a1*^cKO. **(C,E)** Occlusal view of mandibular molars (M_1_, M_2_, M_3_) showing deep molar pits (blue arrowheads) and misaligned teeth (dotted line) in 4 wk old *Trps1*^*Col1a1*^cKO males and females. Scale bar = 10 μm.

Body weight measurements of WT and *Trps1*^*Col1a1*^cKO mice demonstrated that *Trps1*^*Col1a1*^cKO males are significantly smaller than WT littermates at P7, P21 and 4 wks of age. This difference was not found in females, suggesting that *Trps1* deficiency has stronger effect in males than in females (Figure 2A). Gross examination detected no aberrations in the tooth number in *Trps1*^*Col1a1*^cKO and no apparent differences in the tooth crown morphology compared to WT mice. However, analysis of the dental phenotype under a dissecting microscope showed that teeth of 4 wk old mice were “chalky” white and opaque in contrast to the translucent aspect observed in molars of WT mice, irrespective of the sex (Figures 2B–E). Such “chalky” appearance of teeth suggests hypomineralization of dental tissues (66, 67). Additionally, both *Trps1*^*Col1a1*^cKO male and female mice presented severe root exposure at 4 wks of age, suggesting impaired formation of the alveolar bone (Figures 2B,D). Misalignment of the molars was also detected in *Trps1*^*Col1a1*^cKO males and females. Furthermore, more detailed microscopic examination of the occlusal surface of mandibular molars revealed deep pits in *Trps1*^*Col1a1*^cKO mice (Figures 2C,E; blue arrowheads), which suggests defects in crown mineralized tissues formation.

### *Trps1* Deficiency in Odontoblasts Impairs Formation of Dental Mineralized Tissues

**FIGURE 3 |:**
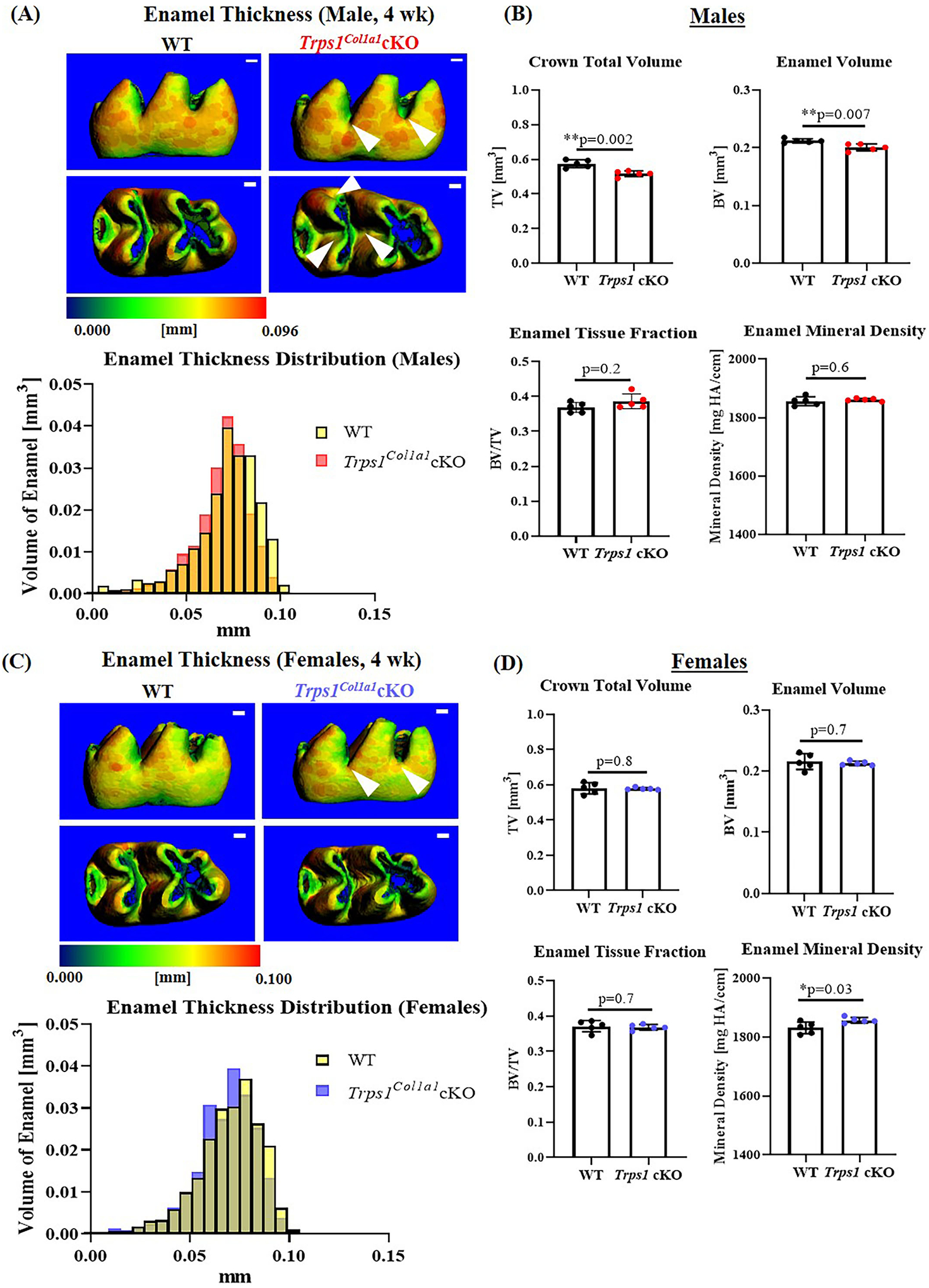
Quantitative μCT analyses of the enamel of mandibular first molars of 4 wk old WT and *Trps1*^*Col1a1*^cKO mice. **(A,C)** Representative 3D μCT images of the enamel; scale bar = 100 μm. The enamel was pseudo-colored based on the distribution of tissue thickness. The corresponding tissue thickness color scale is shown below the images. White arrowheads point at the reduced enamel thickness specifically in pits of *Trps1*^*Col1a1*^cKO male molars. There was no noticeable difference between WT and *Trps1*^*Col1a1*^cKO females. The graph below the 3D images shows the comparison of the quantity (volume) of the enamel with specific thickness in WT and *Trps1*^*Col1a1*^cKO. (B,D) Quantification of enamel volume, enamel tissue fraction in the total crown volume, and mineral density. Mean values ± SD and individual data points (dots, N=5/genotype/sex) are shown; *p < 0.05 and **p < 0.01.

To understand the effect of the odontoblast-specific deficiency of *Trps1* on the quality of the tooth crown, we performed quantitative μCT analyses of enamel and crown dentin volume, tissue thickness, tissue fraction in the total crown volume, and mineral density in mandibular first molars of WT and *Trps1*^*Col1a1*^cKO 4wk old mice. Results of μCT analyses of enamel thickness on smooth surfaces of molars showed no overall difference between WT and *Trps1*^*Col1a1*^cKO mice (Figures 3A,C, top panel images). However, pseudo-coloring of 3D μCT images, based on the tissue thickness in each area, revealed pronounced buccal pits in male and female *Trps1*^*Col1a1*^cKO molars (Figures 3A,C, white arrowheads). Furthermore, reduced enamel thickness in occlusal pits became evident in *Trps1*^*Col1a1*^cKO males (Figure 3A, bottom panel image; Supplementary Figure 2). Interestingly, these areas correspond to locations particularly susceptible to dental caries in humans. The crown total volume and enamel volume were significantly reduced only in *Trps1*^*Col1a1*^cKO males (Figure 3B), suggesting that their teeth are smaller compared to WT. There was no difference in enamel mineral density in males (Figure 3B), but, surprisingly, it was significantly higher in *Trps1*^*Col1a1*^cKO females compared to WT (Figure 3D). Since in *Trps1*^*Col1a1*^cKO mice, *Trps1* is deleted in odontoblasts, and ameloblasts do not express *Trps1* (29), these results suggest that enamel defects in *Trps1*^*Col1a1*^cKO mice result from a cell non-autonomous mechanism.

**FIGURE 4 |:**
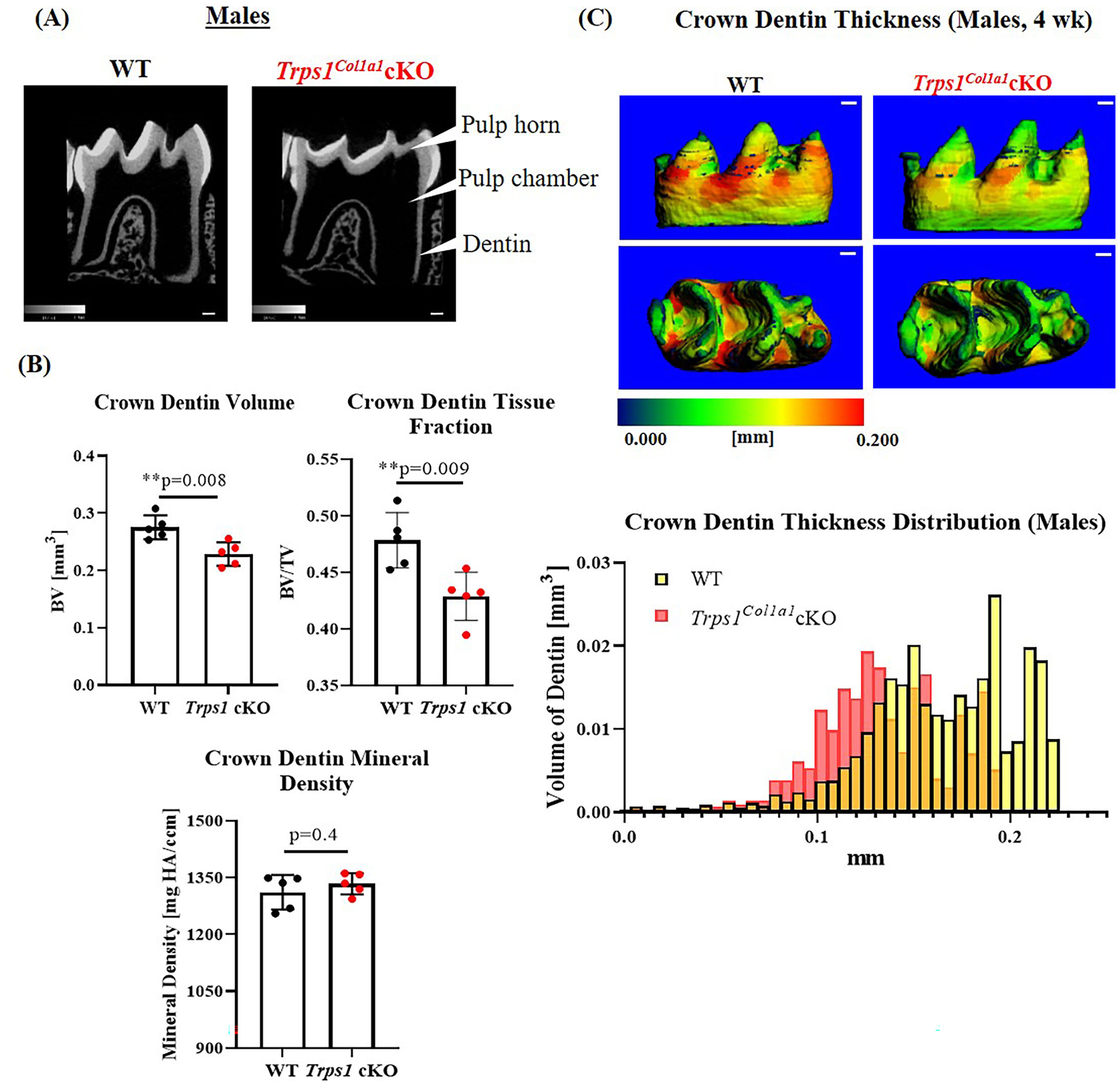
Quantitative μCT analyses of crown dentin of mandibular first molars of 4 wk old WT and *Trps1*^*Col1a1*^cKO males. **(A)** Representative 2D μCT images showing reduced dentin thickness (white arrowheads) and enlarged pulp chambers in *Trps1*^*Col1a1*^cKO males compared to WT. **(B)** Quantification of crown dentin volume, dentin tissue fraction in the total crown volume and mineral density. Mean values ± SD and individual data points (dots, *N* = 5/genotype) are shown; **p < 0.01. **(C)** Representative 3D μCT images of the crown dentin; scale bar = 100 μm. The dentin was pseudo-colored based on the distribution of tissue thickness. The corresponding tissue thickness color scale is shown below the images. Note thinner dentin at the lingual and occlusal surface in *Trps1*^*Col1a1*^cKO males vs. WT. The graph below the 3D images shows the comparison of the quantity (volume) of the crown dentin with specific thickness in WT and *Trps1*^*Col1a1*^cKO males.

**FIGURE 5 |:**
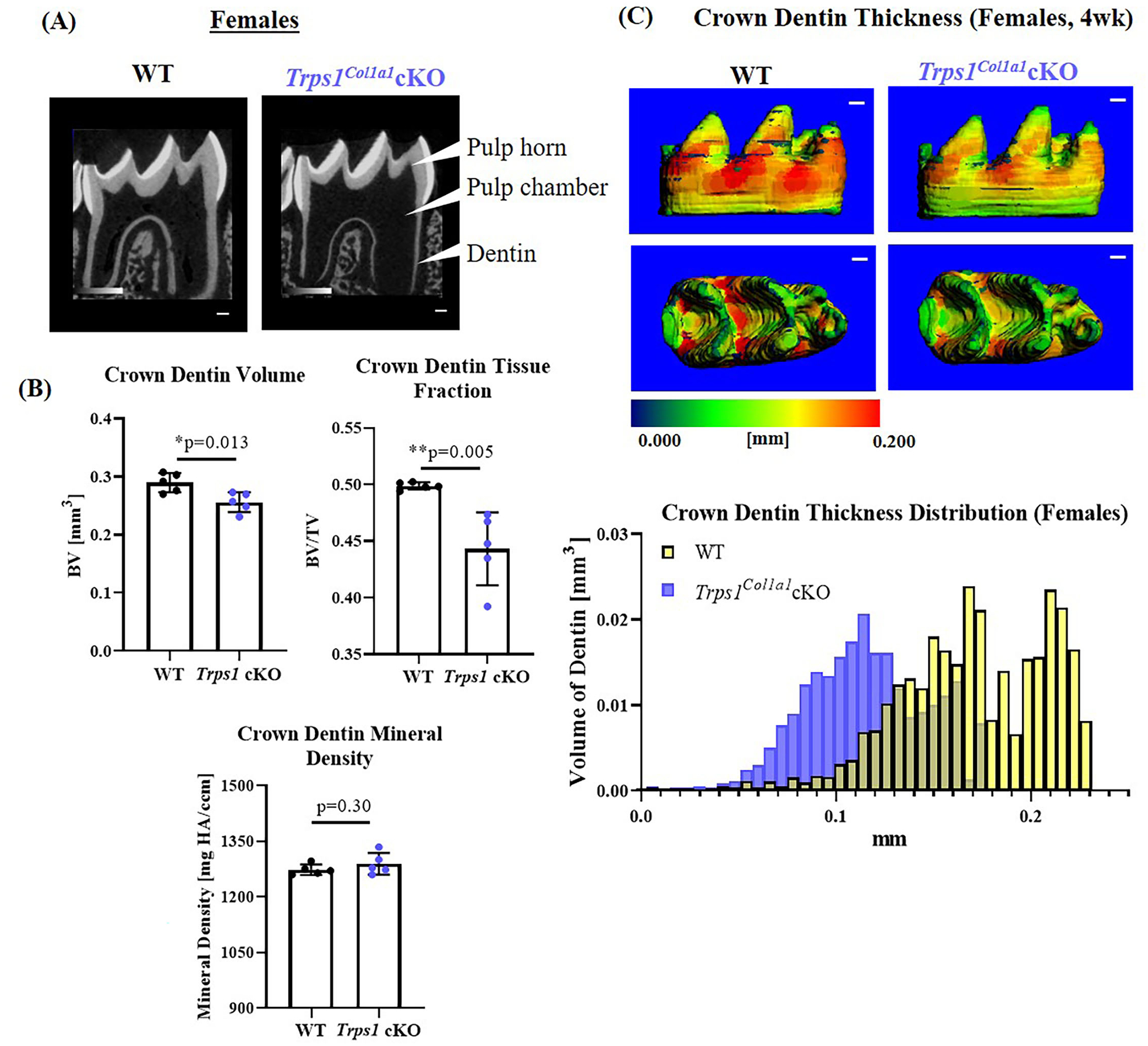
Quantitative μCT analyses of crown dentin of mandibular first molars of 4 wk old WT and *Trps1*^*Col1a1*^cKO females. **(A)** Representative 2D μCT images showing reduced dentin thickness (white arrowheads) and enlarged pulp chambers in *Trps1*^*Col1a1*^cKO females compared to WT. **(B)** Quantification of crown dentin volume, dentin tissue fraction in the total crown volume and mineral density. Mean values ± SD and individual data points (dots, *N* = 5/genotype) are shown; *p < 0.05 and **p < 0.01. **(C)** Representative 3D μCT images of the crown dentin; scale bar = 100 μm. The dentin was pseudo-colored based on the distribution of tissue thickness. The corresponding tissue thickness color scale is shown below the images. The graph below the 3D images shows the comparison of the quantity (volume) of the crown dentin with specific thickness in WT and *Trps1*^*Col1a1*^cKO females.

**FIGURE 6 |:**
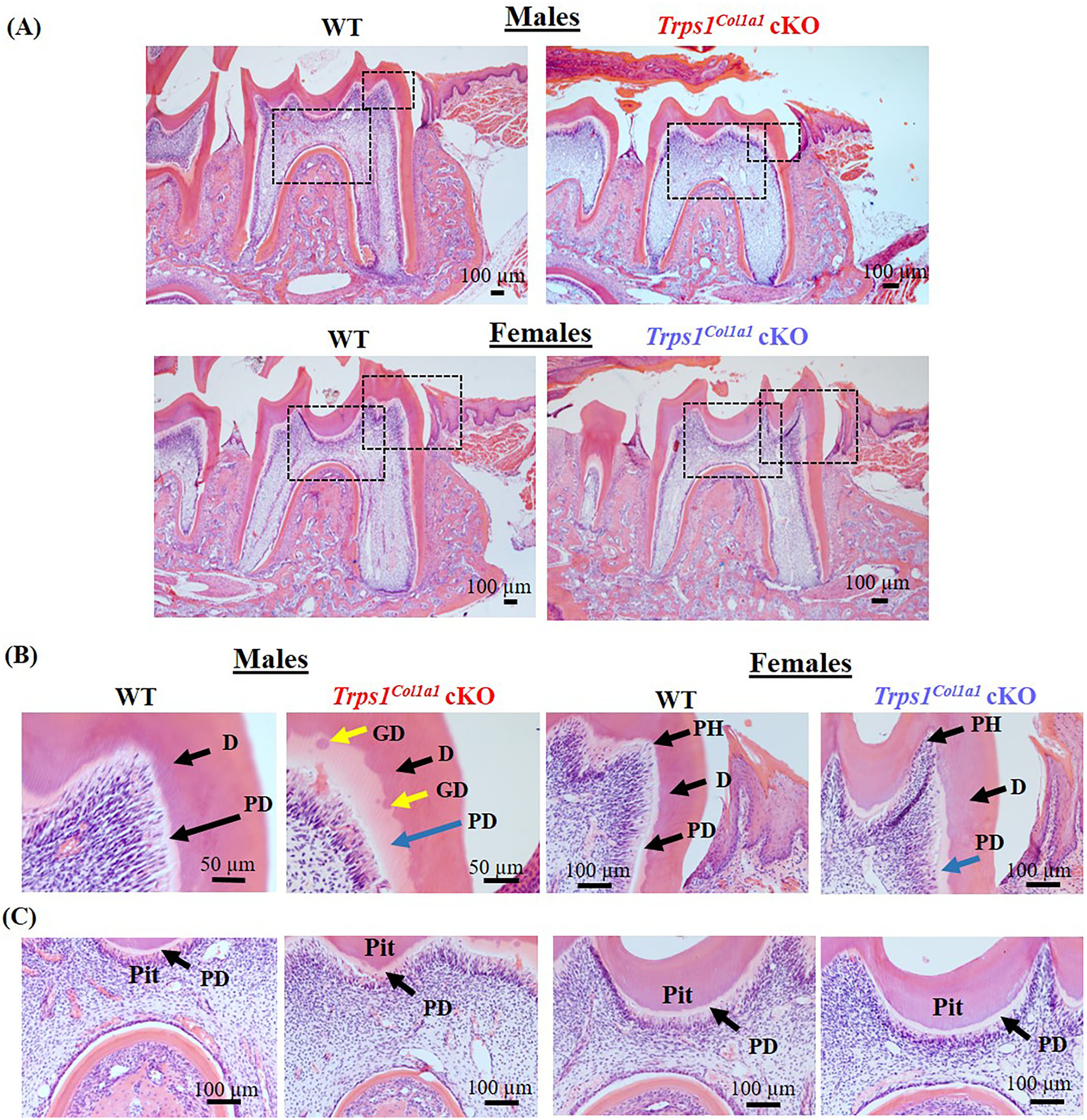
Histological evaluation (H&E staining) of the mandibular first molar crown tissues in 4wk old WT and *Trps1*^*Col1a1*^cKO mice. **(A)** Low magnification images showing gross histological appearance of molars. Boxed areas are shown in higher magnification on panels B and C. **(B)** High magnification of the mesial cusp showing wider predentin in *Trps1*^*Col1a1*^cKO males (blue arrow) and irregular mineralization of crown dentin with calcospherites (globular dentin, yellow arrow). Note the pronounced pulp horns (arrows) in *Trps1*^*Col1a1*^cKO females. **(C)** High magnification of the pit area. Note the deeper appearance of the pit in *Trps1*^*Col1a1*^cKO males. Pulp horn (PH), dentin (D), predentin (PD), globular dentin (GD). *N* = 3 mice/genotype were analyzed.

μCT analyses of crown dentin showed significantly reduced dentin volume and tissue fraction in male and female *Trps1*^*Col1a1*^cKO molars in comparison to WT (Figures 4, 5). Decreased dentin quantity was further demonstrated by pseudo-coloring of 3D μCT images, which showed reduced dentin thickness throughout the crown in *Trps1*^*Col1a1*^cKO males and females (Figures 4C, 5C; Supplementary Figure 3). μCT analyses did not detect significant differences in crown dentin mineral density (Figures 4B, 5B). However, histological analyses of molars uncovered wider predentin, the presence of globular dentin (Figures 6A,B) and pronounced occlusal pits, especially in *Trps1*^*Col1a1*^cKO males (Figures 2C,E, 6C). Furthermore, μCT 2D images (Figures 4A, 5A) revealed enlarged pulp chambers and prominent pulp horns in both *Trps1*^*Col1a1*^cKO male and female mice, while μCT 3D images demonstrated some cases of attrition (Supplementary Figure 4) in *Trps1*^*Col1a1*^cKO mice. In summary, the μCT and histological analyses of molars from 4wk old mice suggest that deficiency of *Trps1* in odontoblasts impairs dentin formation, which, in turn, compromises the enamel.

### Odontoblast-Specific *Trps1* Deficiency Reduces Dentin and Enamel Microhardness and Increases Mineral Loss in Acidic Conditions

**FIGURE 7 |:**
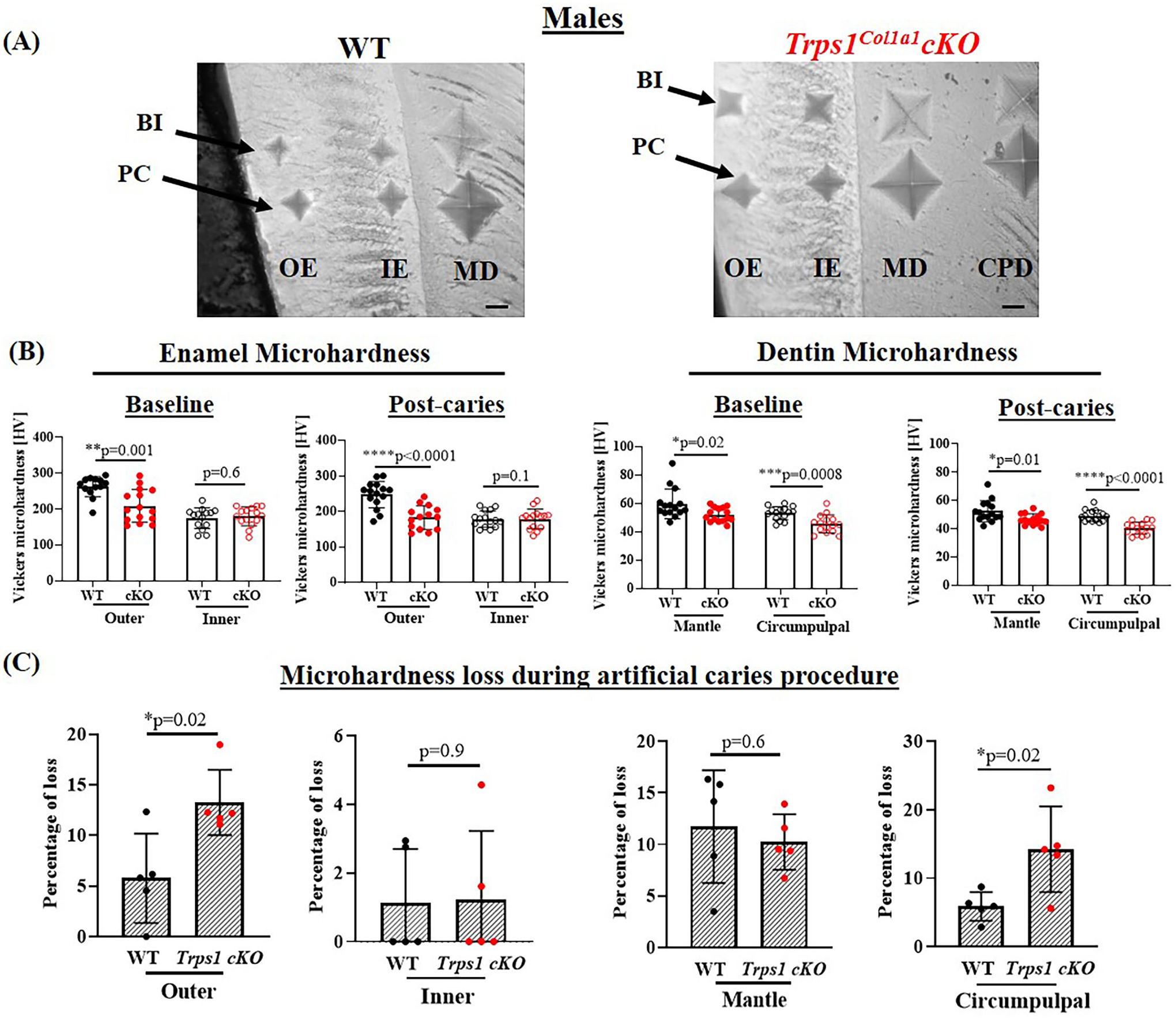
Vickers microhardness analyses of enamel and crown dentin of mandibular first molars in 4 wk old WT and *Trps1*^*Col1a1*^cKO males. **(A)** Light microscopy images showing baseline indentations (BI), and indentations made after the artificial caries procedure (post-caries, PC) in the following regions: outer enamel (OE), inner enamel (IE), crown mantle (MD) and circumpulpal dentin (CPD); scale bar=10 μm. Three indentations (one per occlusal, middle, and cervical third of the crown) were made per region/molar. **(B)** Summary of the results of microhardness analyses of enamel and crown dentin. Data are shown as mean values of mechanical hardness (HV) ± SD and individual data points of all indentations performed in each region (*N* = 5 molars/genotype, 3 indentations/region/molar), *p < 0.05, **p < 0.01, *** *p* < 0.001 and ****p < 0.0001. **(C)** Graphs illustrating the percentage of microhardness loss during the artificial caries procedure. Data are shown as mean values of the % of microhardness loss ± SD and individual data points per mouse molar.

**FIGURE 8 |:**
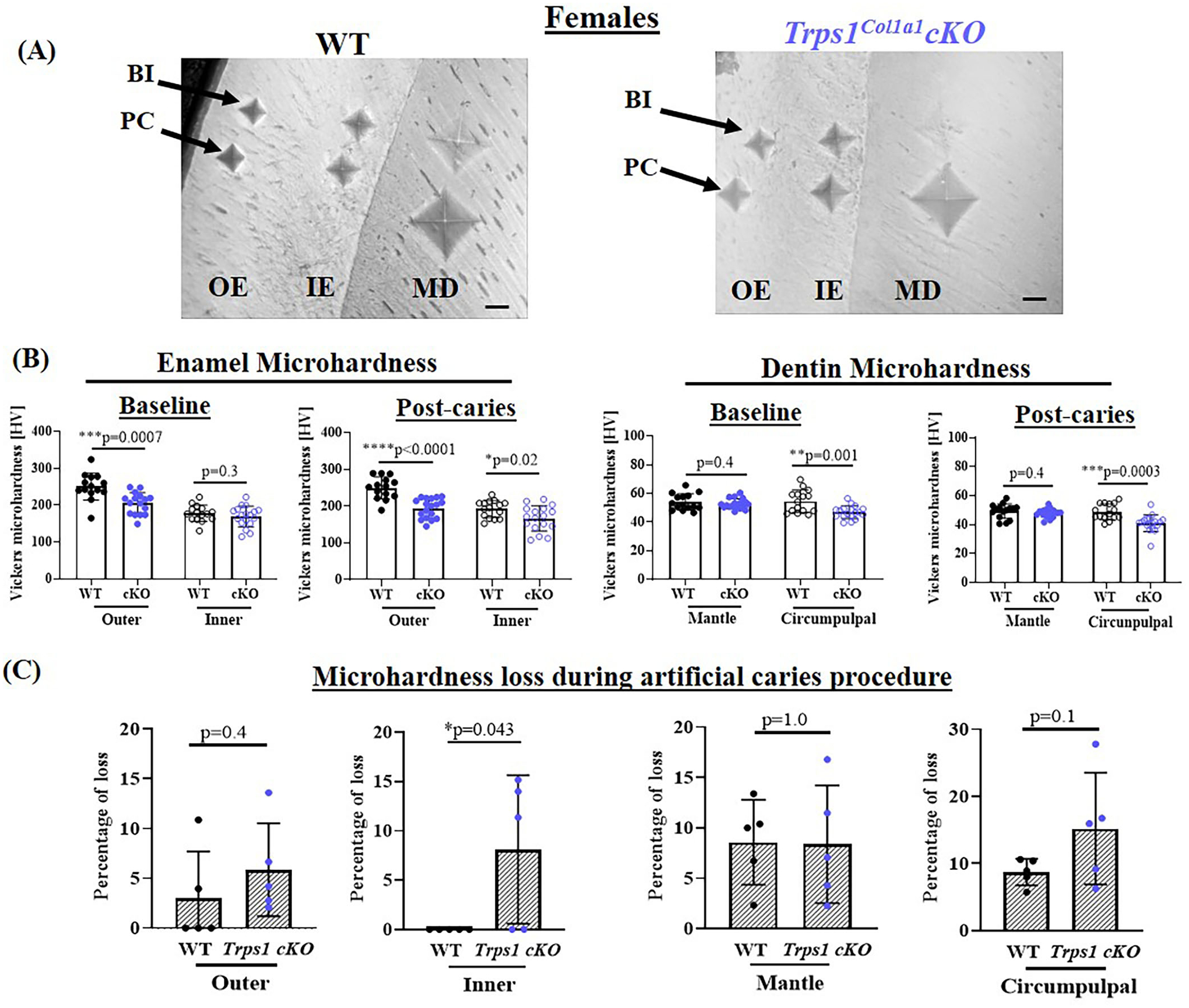
Vickers microhardness analyses of enamel and crown dentin of mandibular first molars in 4 wk old WT and *Trps1*^*Col1a1*^cKO females. **(A)** Light microscopy images showing baseline indentations (BI), and indentations made after the artificial caries procedure (post-caries, PC) in the following regions: outer enamel (OE), inner enamel (IE), crown mantle (MD) and circumpulpal dentin (CPD); scale bar=10 μm. Three indentations (one per occlusal, middle, and cervical third of the crown) were made per region/molar. **(B)** Summary of the results of microhardness analyses of enamel and crown dentin. Data are shown as mean values of mechanical hardness (HV) ± SD and individual data points of all indentations performed in each region (*N* = 5 molars/genotype, 3 indentations/region/molar), *p < 0.05, **p < 0.01, *** *p* < 0.001 and ****p < 0.0001. **(C)** Graphs illustrating the percentage of microhardness loss during the artificial caries procedure. Data are shown as mean values of the % of microhardness loss ± SD and individual data points per mouse molar.

**FIGURE 9 |:**
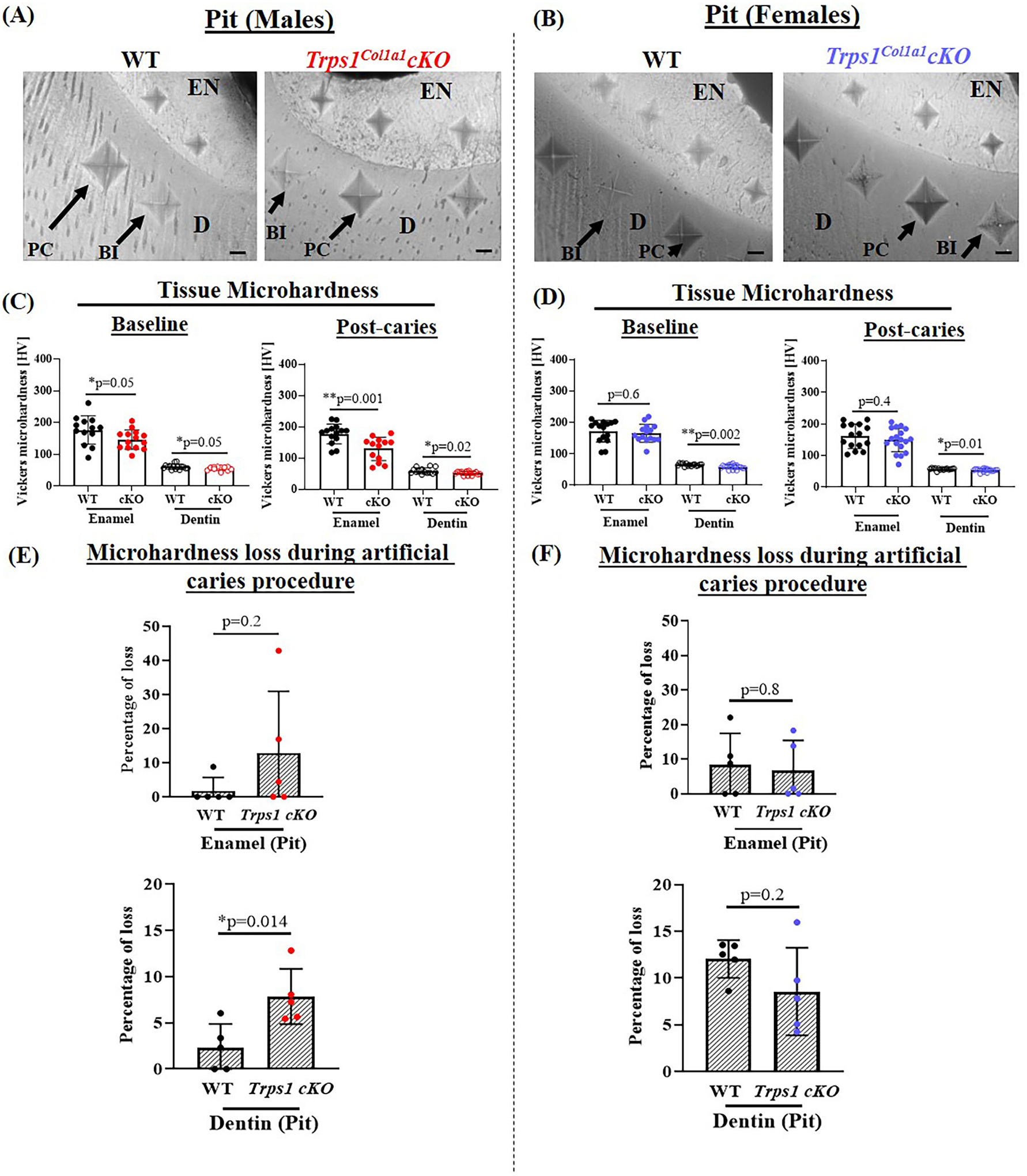
Vickers microhardness analyses of enamel and dentin in the pit of mandibular first molars in 4 wk old WT and *Trps1*^*Col1a1*^cKO male and female mice. Three indentations were made per tissue per molar. **(A,B)** Light microscopy images showing baseline indentations (BI), and indentations made in the enamel (EN) and dentin (D) after the artificial caries procedure (post-caries, PC) in pits; scale bar=10 μm. **(C,D)** Summary of the results of microhardness analyses. Data are shown as values of mechanical hardness (HV) ± SD and individual data points of all indentations performed in each region (*N* = 5 molars/genotype/sex, 3 indentations/tissue/molar), *p < 0.05 and **p < 0.01. **(E,F)** Graphs illustrating the percentage of microhardness loss during the artificial caries procedure. Data are shown as mean values of the % of microhardness loss ± SD and individual data points per mouse molar.

To determine whether the deficiency of *Trps1* in odontoblasts affects the quality of dental tissues, we analyzed enamel and crown dentin hardness in 4 wk old WT and *Trps1*^*Col1a1*^cKO male and female mice using the Vickers microhardness test. We analyzed separately outer and inner enamel, and mantle and circumpulpal dentin. The images of the indentations performed with a Vickers diamond in the enamel and dentin illustrate differences between WT and *Trps1*^*Col1a1*^cKO tissues (Figures 7A, 8A, 9A,B). Results of Vickers microhardness measurements (HV values) demonstrated that odontoblast-specific *Trps1* deficiency resulted in formation of softer (lower HV values) enamel and dentin (Figures 7, 8; Table 1). Specifically, the outer enamel and circumpulpal dentin were significantly softer at the baseline in *Trps1*^*Col1a1*^cKO males and females than in WT mice (Figures 7B, 8B; Table 1).

Since dental caries is caused by the release of organic acids from fermentative bacteria, which results in the dissolution of hydroxyapatite from enamel and dentin (4), our next step was to determine whether the quality of *Trps1*^*Col1a1*^cKO enamel and dentin affects their susceptibility to acid-induced demineralization. For that, we specifically selected an *ex vivo* artificial caries approach to eliminate multiple variables contributing to development of dental caries and specifically focus on quality of dental tissues. To determine the effectiveness of the artificial caries procedure, we did two different types of analyses. First, we compared HV values at baseline and post-demineralization in each tissue (Table 1). Second, we calculated the percentage of microhardness loss during the artificial caries procedure in each analyzed tissue (Figures 7C, 8C, 9E,F; Table 1).

After acid-induced demineralization, in WT mice, only dentin microhardness was significantly lower compared to baseline, while in *Trps1*^*Col1a1*^cKO mice both dentin and enamel were significantly affected (Table 1). This demonstrated that the *ex vivo* demineralization procedure was effective. More importantly, these analyses revealed that *Trps1*^*Col1a1*^cKO tissues were more prone to acid-induced demineralization than WT tissues. This is further highlighted by the calculations of the percentage of microhardness loss, which revealed stronger demineralization (loss of hardness) specifically in the circumpulpal dentin, pit dentin and outer enamel in *Trps1*^*Col1a1*^cKO males (Figures 7C, 9E; Table 1); as well as in the inner enamel in *Trps1*^*Col1a1*^cKO females in comparison with WT mice (Figure 8C).

In the molar pits (Figure 9), enamel of *Trps1*^*Col1a1*^cKO males had significantly lower HV values than in WT males and *Trps1*^*Col1a1*^cKO females at baseline and post-acid demineralization (Figures 9C,D; Table 1), demonstrating overall softer tissues in the pits of *Trps1*^*Col1a1*^cKO male molars.

In summary, results of the microhardness analyses at baseline and after acid-induced demineralization demonstrate that odontoblast-specific *Trps1* deficiency not only decreases enamel and crown dentin microhardness, but also results in formation of dental tissues that are less resistant to demineralization in acidic conditions.

## DISCUSSION

The goal of this study was to provide insights into the pathology underlying the increased susceptibility to dental caries phenotype in TRPS patients (36, 44). Considering that: (i) *Trps1* is expressed in odontoblasts (29, 30, 46), (ii) *Trps1* haploinsufficiency impairs tooth mineralization in mice (30), and (iii) deficiency in odontoblast cell line reduces expression of multiple mineralization-related genes (47), we focused on the role of *Trps1* in the odontoblast function—formation of dentin, and the consequences of *Trps1* deficiency on the quality of the tooth crown tissues. For that, we generated a new animal model—*Trps1*^*Col1a1*^cKO mice, in which *Trps1* is deleted in odontoblasts. Our μCT analyses of mouse molars revealed that odontoblast-specific deletion of *Trps1* leads to decreased crown dentin volume, while no significant differences were detected in dentin mineral density. However, histological analyses revealed a wider predentin and globular dentin pattern in *Trps1*^*Col1a1*^cKO mice, which are indicative of disturbed dentin mineralization (68). Interestingly, the functional *ex vivo* assays assessing dental tissues hardness and their susceptibility to acid-induced demineralization revealed that not only dentin, but also enamel hardness was decreased in *Trps1*^*Col1a1*^cKO mice, with some sex-specific differences. Together, the analyses of *Trps1*^*Col1a1*^cKO mice suggest that compromised quality of dental tissues as well as decreased dentin thickness, particularly in the tooth crown regions susceptible to caries, contribute to a high prevalence of dental caries in TRPS patients.

Whether or not an individual experiences dental caries depends on a large range of endogenous factors (e.g., enamel quality, tooth morphology, and saliva composition and flow rate); behavioral features (e.g., diet and oral hygiene); socioeconomic and demographic factors (e.g., age, sex, race); environmental exposures (e.g., oral bacteria and fluoride); along with host-genetics (11, 13, 18, 69). Candidate genes associated with dental caries reported so far include: immune response genes, genes related to taste, those related to saliva flow rates and composition, as well as genes involved in tooth formation and mineralization (17). Using the cKO approach with *Trps1* deletion only in developed odontoblasts, we focused on investigating the role of Trps1 transcription factor as potential contributor to susceptibility to dental caries. Our analyses revealed several characteristics of *Trps1*^*Col1a1*^cKO teeth that may render them more prone to the initiation and progression of dental caries.

First, quantitative μCT analyses demonstrated that dentin in *Trps1*^*Col1a1*^cKO molars is thinner than in WT mice. This deficiency was particularly prominent in pits of the tooth crown, which are the sites most susceptible to dental caries initiation in humans. Teeth with thinner dentin may be prone to more severe caries, as once a lesion is initiated, it can reach the pulp faster than in teeth with a thicker dentin layer. On radiographic images of teeth, features such as enlarged pulp chambers and prominent pulp horns suggest a thinner dentin layer. This was visible on 2D μCT images of *Trps1*^*Col1a1*^cKO molars, and importantly, such features were recently reported in permanent molars of a 16-year-old male patient with *TRPS1* mutation (32). The authors of this report proposed that large pulp chambers in this individual are a consequence of the *TRPS1* mutation. The detection of the same characteristics in *Trps1*^*Col1a1*^cKO molars supports this conclusion and ratifies *Trps1*^*Col1a1*^cKO mice as a suitable animal model for studying Trps1 function in odontoblasts and molecular pathologies underlying some of the dental phenotypes in TRPS.

Second, tissue microhardness and *ex vivo* artificial caries experiments uncovered that both dentin as well as enamel of *Trps1*^*Col1a1*^cKO teeth are more prone to acid-induced demineralization than WT tissues. This suggests that in TRPS patients, acid produced by oral bacterial fermentation of dietary carbohydrates can destroy dental tissues easier than in healthy individuals, hence making them more prone to the initiation of dental caries as well as enables caries progression. Formation of teeth which are softer and more susceptible to acid demineralization in *Trps1*^*Col1a1*^cKO mice, along with thinner dentin, strongly suggests that compromised quality of dental tissues contributes to increased incidence and severity of dental caries in TRPS patients.

Interestingly, and highly relevant to the development of dental caries, the enamel quality is also impaired by *Trps1* deficiency in odontoblasts, as evident from lower enamel microhardness, and some cases of attrition in *Trps1*^*Col1a1*^cKO mice. The outer enamel (OE) of both *Trps1*^*Col1a1*^cKO males and females is significantly softer, than in the WT mice; while the inner enamel (IE) hardness of *Trps1*^*Col1a1*^cKO females becomes significantly lower after the artificial caries procedure as compared to WT. Notably, the detection of localized enamel mineralization defects in pits contributes to our understanding of dental caries lesion distribution pattern in TRPS. We agree with others, that the effects of dental caries risk factors may be surface-specific (10). Most likely, in *Trps1*^*Col1a1*^cKO mice, the enamel mineral maturation stage, and not the secretory ameloblast functions, are compromised, since no difference in the enamel thickness was detected in most of the surfaces of the first molars. The effect of the odontoblast-specific deletion of *Trps1* on the enamel is not surprising, as there is evidence that formation of enamel is influenced by developing dentin. The importance of proper dentin development for formation of enamel is underscored by severe enamel defects in genetic dentin mineralization disorders such as dentinogenesis imperfecta II, and hypophosphatemic rickets (19, 70–72). Additionally, the differentiation and function of ameloblasts is regulated by signaling from odontoblasts (73). For example, hedgehog and Wnt signaling pathways, which are implicated in molecular communication between developing odontoblasts and ameloblasts, have been shown to be regulated by Trps1 in developing bones and hair follicles (34, 50, 74–76). Hence, these signaling pathways may be disturbed in *Trps1*-deficient odontoblasts, affecting ameloblast function and enamel formation. Furthermore, Trps1 is essential for odontoblast ability to release matrix vesicles (MVs), as shown in our previous *in vitro* studies (47). Although, in mineralized tissues, the MVs main function is to support the initiation of mineralization, there is growing evidence that, like other extracellular vesicles, MVs also participate in the intercellular communication (77). Hence, the formation of weaker enamel in *Trps1*^*Col1a1*^cKO mice results from cell non-autonomous mechanisms, secondary to either defective dentin formation or signaling from *Trps1*-deficient odontoblasts.

We did not detect significant differences in dentin and enamel mineral density of *Trps1*^*Col1a1*^cKO molars versus WT, although correlation between microhardness and mineral content has been reported in human enamel (78). However, the mechanical hardness of mineralized tissues does not depend solely on the mineral content; it depends also on the organization of the mineral (hydroxyapatite crystals) in the ECM (79). It is likely then, that *Trps1* deficiency affects the organization of hydroxyapatite crystals but not the mineralization extent. This will be addressed in a follow-up study of the composition and structure of dental tissues in *Trps1*^*Col1a1*^cKO mice.

Consistent with our previous *in vitro* studies (47), we detected impaired initiation of the dentin mineralization in *Trps1* deficiency. This was detected through histological analyses as the presence of globular dentin and enlarged predentin, which are apparent on the microscopic images. The presence of the globular dentin in *Trps1*^*Col1a1*^cKO mice, along with widened predentin and thin mineralized dentin, resembles mineralization defects observed in hypophosphatemic rickets—a rare disease caused by mutations in phosphate homeostasis genes (80). While such dentin mineralization defects in hypophosphatemic rickets are caused by systemic phosphate deficiency (81, 82), our previous studies of Trps1 function in odontoblasts indicate that similar phenotype can be caused by cell autonomous mechanisms. Specifically, we demonstrated that expression of several genes involved in phosphate homeostasis and hypophosphatemic rickets is regulated by Trps1 in odontoblasts (47). Moreover, Trps1 activity in cells producing mineralizing ECM changes depending on the status of the available extracellular phosphate (83), which also may contribute to the dentin mineralization problems in *Trps1*^*Col1a1*^cKO mice. Trps1 was also shown to repress expression of the *Dspp* gene coding for the major ECM protein of dentin (46), while the *Trps1* deficiency in 17IIA11 odontoblast cell line significantly impaired ability of these cells to mineralize, which was accompanied by downregulation of multiple mineralization-related genes (47). Hence, it is likely that impaired dentin formation in *Trps1*^*Col1a1*^cKO mice is caused by dysregulated transcription in odontoblasts that affects multiple genes crucial for the formation of sound dentin.

Interestingly, *Phex* is one of the phosphate homeostasis genes regulated by Trps1 in odontoblasts and is also mutated in the most common form of the hypophosphatemic rickets, the X-linked hypophosphatemic rickets (47, 80, 84, 85). Hypophosphatemia caused by *PHEX* mutation results in a severely disrupted formation of circumpulpal dentin, while mantle dentin is unaffected. Since, all of these dental pathologies are present in *Trps1*^*Col1a1*^cKO mice, affecting especially males; this suggests that differences between dental phenotypes of *Trps1*^*Col1a1*^cKO males and females might be in part due to dysregulation of the *Phex* gene, which is located on the X-chromosome. Another possible explanation of the differences between *Trps1*^*Col1a1*^cKO males and females is that the *Trps1* itself has been reported to interact with sex hormones such as androgen and estrogen (86–88). The interaction of Trps1 with sex hormones is quite complex and occurs at multiple levels. For example, human prostate cancer studies demonstrated that *TRPS1* is repressed by androgens. On the other hand, TRPS1 can inhibit androgen receptor signaling driving genes such as prostate-specific antigen and promote apoptosis in the absence of androgen (86, 89, 90). TRPS1 has been also shown to regulate estrogen receptor binding to regulatory DNA elements, in part by modulation of chromatin transcriptional availability (91). The reported here differences between *Trps1*^*Col1a1*^cKO male and female dental mineralized tissues are consistent with the previous reports that Trps1 differentially modulates the bone mineral density between male and female mice (92). This set of evidence may explain in part, why 4 wk old *Trps1*^*Col1a1*^cKO males (during onset of puberty), present with a stronger dental phenotype compared to females.

Among other dental findings in *Trps1*^*Col1a1*^cKO mice relevant to the predisposition to dental caries is malocclusion. Dental caries is a common complication of malocclusion (93). This abnormality, presented as misalignment of the teeth relative to the body of a mandible, was detected in both *Trps1*^*Col1a1*^cKO males and females. This and severe root exposure detected in *Trps1*^*Col1a1*^cKO mice suggest impaired formation of the alveolar bone, which is caused most likely by deficiency of *Trps1* in osteoblasts, as osteoblasts are another cell type expressing Cre from the *2.3kbCol1a1* promoter (54). Notably, severe tooth misalignments not only increase risk of dental caries but also of periodontal disease (94), underlining the role of *Trps1* not only in formation of sound mineralized dental tissues, but also in the dento-alveolar complex.

In summary, the compromised quality of the tooth mineralized tissues, expressed as softer and less acid-resistant enamel and dentin, together with decreased dentin layer, tooth misalignment, localized tooth mineralization defects in occlusal and buccal pits detected in *Trps1*^*Col1a1*^cKO mice suggests that TRPS patients are genetically predisposed to dental caries. Hence, TRPS patients may benefit from more assertive prevention strategies and early interventions to mitigate dental caries risk and improve oral health.

## Supplementary Material

Supplementary Materials

## Figures and Tables

**TABLE 1 |:** Summary of Vickers microhardness analyses and effect of artificial caries procedure on tissue microhardness loss in WT and *Trps1*^*Col1a1*^cKO mandibular first molars.

	Region	Baseline [HV]		Post-Artificial Caries [HV]		% Microhardness loss	
		WT	*Trps1*^*Col1a1*^cKO	WT	*Trps1*^*Col1a1*^cKO	WT	*Trps1*^*Col1a1*^cKO
Males	Outer Enamel (OE)	**263.3 ± 16.4**	**208.8 ± 27.6** *	**251.0 ± 28.5**	181.5 ± 27.6** (^†††^)	**5.8 ± 5.1**	**13.3 ± 3.2** *
	Inner Enamel (IE)	175.5 ± 14.2	179.3 ± 22.6	178.1 ± 18.8	178.3 ± 22.5	1.1 ± 1.6	1.2 ± 2.0
	Mantle Dentin (MD)	59.7 ± 7.7	52.0 ± 3.9	**52.5 ± 4.4**(^†^)	46.6 ± 2.9* (^††^)	11.7 ± 5.5	10.2 ± 2.7
	Circumpulpal Dentin (CPD)	**52.9 ± 2.5**	**45.6 ± 4.6** *	**49.8 ± 2.7**(^†^)	38.9 ± 2.0*** (^†^)	**5.9 ± 2.4**	**14.2 ± 7.2** *
	Enamel (Pit)	**180.6 ± 19.7**	**147.2 ± 18.3** *	**186.5 ± 7.8**	**130.7 ± 30.5** **	2.2 ± 4.4	12.8 ± 18.2
	Dentin (Pit)	60.5 ± 9.2	55.8 ± 5.3	59.7 ± 10.4	51.5 ± 5.8 (^††^)	**2.3 ± 2.9**	**7.8 ± 3.0** *
Females	Outer Enamel (OE)	**250.7 ± 22.8**	**204.4 ± 20.8** *	**248.9 ± 17.1**	193.1 ± 27.6** (^†^)	3.0 ± 4.7	5.9 ± 4.7
	Inner Enamel (IE)	177.6 ± 15.8	173.1 ± 16.1	191.9 ± 18.7	169.3 ± 33.4	**0.0**	**8.1 ± 7.5** *
	Mantle Dentin (MD)	54.0 ± 5.8	52.6 ± 3.3	49.3 ± 4.3(^†^)	48.0 ± 2.5 (^†^)	8.6 ± 4.2	8.4 ± 5.8
	Circumpulpal Dentin (CPD)	**54.2 ± 5.5**	**47.2 ± 3.0** *	**49.5 ± 4.9**(^††^)	40.0 ± 4.4* (^†^)	8.7 ± 2.0	15.2 ± 8.3
	Enamel (Pit)	172.1 ± 24.4	153.6 ± 33.4	161.1 ± 39.2	146.6 ± 37.3	8.4 ± 9.13	6.7 ± 8.7
	Dentin (Pit)	65.1 ± 3.0	58.1 ± 6.8	57.3 ± 2.7(^†††^)	52.9 ± 4.7 (^†^)	12.0 ± 2.0	8.6 ± 4.7

The data are expressed as the mean values ± SD (N = 5 mice per group; for each mouse the mean value of 3 indentations in each tissue was used). Mechanical hardness values and the percentage of microhardness loss during the artificial caries procedure were compared between WT and Trps1^Col1a1^cKO using an unpaired t-test.

* p < 0.05,

** p < 0.01,

*** p < 0.001.

Bold numbers highlight statistical significant differences between WT and Trps1^Col1a1^cKO. Comparison (paired t-test) between baseline and post-artificial caries [HV] of outer enamel (OE), inner enamel (IE), mantle (M), and circumpulpal dentin (CPD) /genotype/sex,

† p<0.05,

†† p< 0.01,

††† p<0.001.

## Data Availability

The raw data supporting the conclusions of this article will be made available by the authors, without undue reservation.

## References

[1] Petersen PE, Bourgeois D, Ogawa H, Estupinan-Day S, Ndiaye C. The global burden of oral diseases and risks to oral health. Bull World Health Organ. (2005) 83:661–9. 16211157 PMC2626328

[2] Dye BA. The global burden of oral disease: research and public health significance. J Dent Res. (2017) 96:361–3. doi: 10.1177/0022034517693567 28318392 PMC6728669

[3] Bagramian RA, Garcia-Godoy F, Volpe AR. The global increase in dental caries. a pending public health crisis. Am J Dent. (2009) 22:3–8. 19281105

[4] Pitts NB, Zero DT, Marsh PD, Ekstrand K, Weintraub JA, Ramos-Gomez F, Dental caries. Nat Rev Dis Primers. (2017) 3:17030. doi: 10.1038/nrdp.2017.30 28540937

[5] Fontana M, Young DA, Wolff MS, Pitts NB, Longbottom C. Defining dental caries for 2010 and beyond. Dent Clin North Am. (2010) 54:423–40. doi: 10.1016/j.cden.2010.03.007 20630187

[6] Featherstone JD. The science and practice of caries prevention. J Am Dent Assoc. (2000) 131:887–99. doi: 10.14219/jada.archive.2000.0307 10916327

[7] Manji F, Dahlen G, Fejerskov O. Caries and periodontitis: contesting the conventional wisdom on their aetiology. Caries Res. (2018) 52:548–64. doi: 10.1159/000488948 29694978

[8] Gati D, Vieira AR. Elderly at greater risk for root caries: a look at the multifactorial risks with emphasis on genetics susceptibility. Int J Paediatr Dent. (2011) 2011:647168. doi: 10.1155/2011/647168 PMC3133477 21754932

[9] Demirci M, Tuncer S, Yuceokur AA. Prevalence of caries on individual tooth surfaces and its distribution by age and gender in university clinic patients. Eur J Dent. (2010) 4: 270–9. doi: 10.1055/s-0039-1697839 20613915 PMC2897860

[10] Shaffer JR, Wang X, Desensi RS, Wendell S, Weyant RJ, Cuenco KT, Genetic susceptibility to dental caries on pit and fissure and smooth surfaces. Caries Res. (2012) 46:38–46. doi: 10.1159/000335099 22286298 PMC3304515

[11] Hunter PB. Risk factors in dental caries. Int Dent J. (1988) 38:211–7. 3063664

[12] Shaffer JR, Polk DE, Feingold E, Wang X, Cuenco KT, Weeks DE, Demographic, socioeconomic, and behavioral factors affecting patterns of tooth decay in the permanent dentition: principal components and factor analyses. Community Dent Oral Epidemiol. (2013) 41:364–73. doi: 10.1111/cdoe.12016 23106439 PMC3568445

[13] Shaffer JR, Wang X, McNeil DW, Weyant RJ, Crout R, Marazita ML. Genetic susceptibility to dental caries differs between the sexes: a family-based study. Caries Res. (2015) 49:133–40. doi: 10.1159/000369103 25612913 PMC4449725

[14] Werneck RI, Mira MT, Trevilatto PC. A critical review: an overview of genetic influence on dental caries. Oral Dis. (2010) 16:613–23. doi: 10.1111/j.1601-0825.2010.01675.x 20846151

[15] Werneck RI, Lázaro FP, Cobat A, Grant AV, Xavier MB, Abel L, A major gene effect controls resistance to caries. J Dent Res. (2011) 90:735–9. doi: 10.1177/0022034510397614 21364090 PMC3318028

[16] Opal S, Garg S, Jain J, Walia I. Genetic factors affecting dental caries risk. Aust Dent J. (2015) 60:2–11. doi: 10.1111/adj.12262 25721273

[17] Vieira AR, Modesto A, Marazita ML. Caries: review of human genetics research. Caries Res. (2014) 48:491–506. doi: 10.1159/000358333 24853115 PMC4167926

[18] Shuler CF. Inherited risks for susceptibility to dental caries. Eur J Dent Educ. (2001) 65:1038–45. doi: 10.1002/j.0022-0337.2001.65.10.tb03447.x 11699975

[19] Seow W Developmental defects of enamel and dentine: challenges for basic science research and clinical management. Aust Dent J. (2014) 59:143–54. doi: 10.1111/adj.12104 24164394

[20] Eberle F, Hartenfels S, Pralle H, Käbisch A. Adult hypophosphatasia without apparent skeletal disease: “odontohypophosphatasia” in four heterozygote members of a family. Klin Wochenschr. (1984) 62:371–6. doi: 10.1007/BF01716257 6727276

[21] Margolis HC, Kwak SY, Yamazaki H. Role of mineralization inhibitors in the regulation of hard tissue biomineralization: relevance to initial enamel formation and maturation. Front Physiol. (2014) 5: 339. doi: 10.3389/fphys.2014.00339 25309443 PMC4159985

[22] Giacaman RA, Perez VA, Carrera CA. 5 - Mineralization processes in hard tissues: teeth. In: Aparicio C, Ginebra MP, editors. Biomineralization and Biomaterials. Boston: Woodhead Publishing (2016). p. 147–85.

[23] Boskey AL. The role of extracellular matrix components in dentin mineralization. Crit Rev Oral Biol Med. (1991) 2:369–87. doi: 10.1177/10454411910020030501 1654141

[24] Chen S, Gluhak-Heinrich J, Wang YH, Wu YM, Chuang HH, Chen L, Runx2, Osx, and Dspp in tooth development. J. Dent. Res (2009) 88: 904–09. doi: 10.1177/0022034509342873 19783797 PMC3045537

[25] Bae JM, Clarke JC, Rashid H, Adhami MD, McCullough K, Scott JS, Specificity protein 7 is required for proliferation and differentiation of ameloblasts and odontoblasts. J Bone Miner Res. (2018) 33:1126–40 doi: 10.1002/jbmr.3401 29405385 PMC6002875

[26] Chu Q, Gao Y, Gao X, Dong Z, Song W, Xu Z, Ablation of Runx2 in ameloblasts suppresses enamel maturation in tooth development. Sci Rep. (2018) 8:9594. doi: 10.1038/s41598-018-27873-5 29941908 PMC6018461

[27] Shungin D, Haworth S, Divaris K, Agler CS, Kamatani Y, Keun Lee M, Genome-wide analysis of dental caries and periodontitis combining clinical and self-reported data. Nat Commun. (2019) 10:2773. doi: 10.1038/s41467-019-10630-1 31235808 PMC6591304

[28] Shaffer JR, Feingold E, Wang X, Lee M, Tcuenco K, Weeks DE, GWAS of dental caries patterns in the permanent dentition. J Dent Res. (2013) 92:38–44. doi: 10.1177/0022034512463579 23064961 PMC3521449

[29] Kantaputra P, Miletich I, Lüdecke HJ, Suzuki EY, Praphanphoj V, Shivdasani R, Tricho-Rhino-Phalangeal syndrome with supernumerary teeth. J Dent Res. (2008) 87:1027–31. doi: 10.1177/154405910808701102 18946009

[30] Goss M, Socorro M, Monier D, Verdelis K, Napierala D. Trps1 transcription factor regulates mineralization of dental tissues and proliferation of tooth organ cells. Mol Genet Metab. (2019) 126:504–12. doi: 10.1016/j.ymgme.2019.01.014 30691926 PMC6535116

[31] Momeni P, Glöckner G, Schmidt O, von Holtum D, Albrecht B, Gillessen-Kaesbach G, Mutations in a new gene, encoding a zinc-finger protein, cause tricho-rhino-phalangeal syndrome type I. Nat Genet. (2000) 24:71–4. doi: 10.1038/71717 10615131

[32] Kantaputra PN, Coury SA, Tan WH. Impaired dentin mineralization, supernumerary teeth, hypoplastic mandibular condyles with long condylar necks, and a TRPS1 mutation. Arch Oral Biol. (2020) 116: 104735. doi: 10.1016/j.archoralbio.2020.104735 32442662

[33] Maas SM, Shaw AC, Bikker H, Lüdecke HJ, van der Tuin K, Badura-Stronka M, Phenotype and genotype in 103 patients with tricho-rhino-phalangeal syndrome. Eur J Med Genet. (2015) 58:279–92. doi: 10.1016/j.ejmg.2015.03.002 25792522

[34] Napierala D, Sam K, Morello R, Zheng Q, Munivez E, Shivdasani RA, Uncoupling of chondrocyte differentiation and perichondrial mineralization underlies the skeletal dysplasia in tricho-rhino-phalangeal syndrome. Hum Mol Genet. (2008) 17:2244–54. doi: 10.1093/hmg/ddn125 18424451 PMC2710999

[35] Lüdecke HJ, Schaper J, Meinecke P, Momeni P, Gross S, von Holtum D, Genotypic and phenotypic spectrum in tricho-rhino-phalangeal syndrome types I and III. Am J Hum Genet. (2001) 68:81–91. doi: 10.1086/316926 11112658 PMC1234936

[36] Bennett CG, Hill CJ, Frias JL. Facial and oral findings in trichorhinophalangeal syndrome type 1 (characteristics of TRPS 1). Pediatr Dent. (1981) 3:348–52. 6952172

[37] Stagi S, Bindi G, Galluzzi F, Lapi E, Salti R, Chiarelli F. Partial growth hormone deficiency and changed bone quality and mass in type I trichorhinophalangeal syndrome. Am J Med Genet A. (2008) 146a: 1598–604. doi: 10.1002/ajmg.a.32348 18478599

[38] Macchiaiolo M, Mennini M, Digilio MC, Buonuomo PS, Lepri FR, Gnazzo M, Thricho-rhino-phalangeal syndrome and severe osteoporosis: a rare association or a feature? an effective therapeutic approach with biphosphonates. Am J Med Genet A. (2014) 164a:760–3. doi: 10.1002/ajmg.a.36327 24357341

[39] Niikawa N, Kamei T. The Sugio-Kajii syndrome, proposed tricho-rhino-phalangeal syndrome type III. Am J Med Genet. (1986) 24:759–60. doi: 10.1002/ajmg.1320240420 3740106

[40] Lüdecke HJ, Wagner MJ, Nardmann J, La Pillo B, Parrish JE, Willems PJ, Molecular dissection of a contiguous gene syndrome: localization of the genes involved in the Langer-Giedion syndrome. Hum Mol Genet. (1995) 4:31–6. doi: 10.1093/hmg/4.1.31 7711731

[41] Chen CP, Lin MH, Chen YY, Chern SR, Chen YN, Wu PS, Prenatal diagnosis and array comparative genomic hybridization characterization of interstitial deletions of 8q23.3-q24.11 and 8q24.13 associated with Langer-Giedion syndrome, cornelia de lange syndrome and haploinsufficiency of TRPS1, RAD21 and EXT1. Taiwan J Obstet Gynecol. (2015) 54:592–6. doi: 10.1016/j.tjog.2015.08.013 26522117

[42] Kajii T, Fernandez Gonzalez I, Matsuura S. Tricho-rhino-phalangeal syndrome type III. Am J Med Genet. (1994) 49: 349–50. doi: 10.1002/ajmg.1320490323 8209900

[43] Nagai T, Nishimura G, Kasai H, Hasegawa T, Kato R, Ohashi H, Another family with tricho-rhino-phalangeal syndrome type III (Sugio-Kajii syndrome). Am J Med Genet. (1994) 49:278–80. doi: 10.1002/ajmg.1320490307 8209886

[44] Machuca G, Martínez F, Machuca C, Bullón P. Craniofacial and oral manifestations of trichorhinophalangeal syndrome type I (Giedion’s syndrome): a case report. Oral Surg Oral Med Oral Pathol Oral Radiol Endod. (1997) 84:35–9. doi: 10.1016/S1079-2104(97)90291-2 9247947

[45] Morioka D, Hosaka Y. Aesthetic and plastic surgery for trichorhinophalangeal syndrome. Aesthetic Plast. Surg (2000) 24:39–45. doi: 10.1007/s002669910008 10742468

[46] Napierala D, Sun Y, Maciejewska I, Bertin TK, Dawson B, D’Souza R, Transcriptional repression of the Dspp gene leads to dentinogenesis imperfecta phenotype in Col1a1-Trps1 transgenic mice. J. Bone Miner. Res (2012) 27:1735–45. doi: 10.1002/jbmr.1636 22508542 PMC3399940

[47] Kuzynski M, Goss M, Bottini M, Yadav MC, Mobley C, Winters T, Dual role of the Trps1 transcription factor in dentin mineralization. *Int* J Biol Chem. (2014) 289:27481–93. doi: 10.1074/jbc.M114.550129 25128529 PMC4183789

[48] Malik TH, von Stechow D, Bronson RT, Shivdasani RA. Deletion of the GATA domain of TRPS1 causes an absence of facial hair and provides new insights into the bone disorder in inherited tricho-rhino-phalangeal syndromes. Mol Cell Biol. (2002) 22:8592–600. doi: 10.1128/MCB.22.24.8592-8600.2002 12446778 PMC139891

[49] Suemoto H, Muragaki Y, Nishioka K, Sato M, Ooshima A, Itoh S, Trps1 regulates proliferation and apoptosis of chondrocytes through Stat3 signaling. Dev Biol. (2007) 312:572–81. doi: 10.1016/j.ydbio.2007.10.001 17997399

[50] Wuelling M, Kaiser FJ, Buelens LA, Braunholz D, Shivdasani RA, Depping R, Trps1, a regulator of chondrocyte proliferation and differentiation, interacts with the activator form of gli3. Dev Biol. (2009) 328: 40–53. doi: 10.1016/j.ydbio.2009.01.012 19389374

[51] Cho KY, Kelley BP, Monier D, Lee B, Szabo-Rogers H, Napierala D. Trps1 Regulates development of craniofacial skeleton and is required for the initiation of palatal shelves fusion. Front Physiol. (2019) 10:513. doi: 10.3389/fphys.2019.00513 31130868 PMC6509243

[52] Lungová V, Radlanski RJ, Tucker AS, Renz H, Míšek I, Matalová E. Tooth-bone morphogenesis during postnatal stages of mouse first molar development. J Anat. (2011) 218:699–716. doi: 10.1111/j.1469-7580.2011.01367.x 21418206 PMC3125904

[53] Farley FW, Soriano P, Steffen LS, Dymecki SM. Widespread recombinase expression using FLPeR (flipper) mice. Genesis. (2000) 28: 106–10 11105051

[54] Kim JE, Nakashima K, de Crombrugghe B. Transgenic mice expressing a ligand-inducible cre recombinase in osteoblasts and odontoblasts: a new tool to examine physiology and disease of postnatal bone and tooth. Am J Pathol. (2004) 165:1875–82. doi: 10.1016/S0002-9440(10)63240-3 15579432 PMC1618706

[55] Feil S, Valtcheva N, Feil R. Inducible cre mice. Methods Mol Biol. (2009) 530: 343–63. doi: 10.1007/978-1-59745-471-1_18 19266339

[56] Zhong ZA, Sun W, Chen H, Zhang H, Lay YE, Lane NE, Optimizing tamoxifen-inducible Cre/loxp system to reduce tamoxifen effect on bone turnover in long bones of young mice. Bone. (2015) 81:614–19. doi: 10.1016/j.bone.2015.07.034 26232373 PMC4640982

[57] Couasnay G, Frey C, Elefteriou F. Promoter cre-specific genotyping assays for authentication of cre-driver mouse lines. JBMR Plus. (2019) 3: e10128. doi: 10.1002/jbm4.10128 31044186 PMC6478581

[58] Nariyama M, Shimizu K, Uematsu T, Maeda T. Identification of chromosomes associated with dental caries susceptibility using quantitative trait locus analysis in mice. Caries Res. (2004) 38:79–84. doi: 10.1159/000075929 14767162

[59] Boskey AL, Verdelis K, Spevak L, Lukashova L, Beniash E, Yang X, Mineral and matrix changes in Brtl/+ teeth provide insights into mineralization mechanisms. Biomed Res Int. (2013) 2013:295812. doi: 10.1155/2013/295812 23802117 PMC3681234

[60] Verdelis K, Szabo-Rogers HL, Xu Y, Chong R, Kang R, Cusack BJ, Accelerated enamel mineralization in Dspp mutant mice. Matrix Biol. (2016) 52:246–59. doi: 10.1016/j.matbio.2016.01.003 26780724 PMC4875851

[61] Vieira AR, Gibson CW, Deeley K, Xue H, Li Y. Weaker dental enamel explains dental decay. PloS ONE. (2015) 10:e0124236. doi: 10.1371/journal.pone.0124236 25885796 PMC4401694

[62] Chuenarrom C, Benjakul P, Daosodsai P. Effect of indentation load and time on knoop and vickers microhardness tests for enamel and dentin. Mater Res. (2009) 12:473–76. doi: 10.1590/S1516-14392009000400016

[63] Whitfield J, Littlewood T, Soucek L. Tamoxifen administration to mice. Cold Spring Harb Protoc. (2015) 2015:269–71. doi: 10.1101/pdb.prot077966 25734062 PMC6773604

[64] Indra AK, Warot X, Brocard J, Bornert JM, Xiao JH, Chambon P, Temporally-controlled site-specific mutagenesis in the basal layer of the epidermis: comparison of the recombinase activity of the tamoxifen-inducible Cre-ER(T) and Cre-ER(T2) recombinases. Nucleic Acids Res. (1999) 27:4324–7. doi: 10.1093/nar/27.22.4324 10536138 PMC148712

[65] Valny M, Honsa P, Kirdajova D, Kamenik Z, Anderova M. Tamoxifen in the mouse brain: implications for fate-mapping studies using the tamoxifen-inducible Cre-loxP system. Front Cell Neurosci. (2016) 10: 243. doi: 10.3389/fncel.2016.00243 27812322 PMC5071318

[66] Lacruz RS, Habelitz S, Wright JT, Paine ML. Dental enamel formation and implications for oral health and disease. Physiol Rev. (2017) 97:939–3. doi: 10.1152/physrev.00030.2016 28468833 PMC6151498

[67] Patel A, Aghababaie S, Parekh S. Hypomineralisation or hypoplasia? Br Dent J. (2019) 227:683–6. doi: 10.1038/s41415-019-0782-9 31654000

[68] Clayton D, Chavez MB, Tan MH, Kolli TN, Giovani PA, Hammersmith KJ, Mineralization defects in the primary dentition associated with X-linked hypophosphatemic rickets. JBMR Plus. (2021) 5:e10463. doi: 10.1002/jbm4.10463 33869987 PMC8046057

[69] Wang X, Shaffer JR, Weyant RJ, Cuenco KT, DeSensi RS, Crout R, Genes and their effects on dental caries may differ between primary and permanent dentitions. Caries Res. (2010) 44:277–84. doi: 10.1159/000314676 20516689 PMC2919434

[70] Wright JT, Carrion IA, Morris C. The molecular basis of hereditary enamel defects in humans. J Dent Res. (2015) 94:52–61. doi: 10.1177/0022034514556708 25389004 PMC4270810

[71] Zhang X. Vitamin D Receptor Deficiency and Postnatal Tooth Formation. Birmingham, AL: Citeseer (2007).

[72] Zhang X, Beck P, Rahemtulla F, Thomas HF. Regulation of enamel and dentin mineralization by vitamin D receptor. Front Oral Biol. (2009) 13:102–9. doi: 10.1159/000242400 19828979

[73] He P, Zhang Y, Kim SO, Radlanski RJ, Butcher K, Schneider RA, Ameloblast differentiation in the human developing tooth: effects of extracellular matrices. Matrix Biol. (2010) 29:411–9. doi: 10.1016/j.matbio.2010.03.001 20211728 PMC3296366

[74] Fantauzzo KA, Christiano AM. Trps1 activates a network of secreted Wnt inhibitors and transcription factors crucial to vibrissa follicle morphogenesis. Development. (2012) 139:203–14. doi: 10.1242/dev.069971 22115758 PMC3231778

[75] Wuelling M, Schneider S, Schröther VA, Waterkamp C, Hoffmann D, Vortkamp A. Wnt5a is a transcriptional target of Gli3 and Trps1 at the onset of chondrocyte hypertrophy. Dev Biol. (2020) 457:104–18. doi: 10.1016/j.ydbio.2019.09.012 31550480

[76] Nishioka K, Itoh S, Suemoto H, Kanno S, Gai Z, Kawakatsu M. Trps1 deficiency enlarges the proliferative zone of growth plate cartilage by upregulation of Pthrp. Bone. (2008) 43:64–71. doi: 10.1016/j.bone.2008.03.009 18456591

[77] Ansari S, de Wildt BWM, Vis MAM, de Korte CE, Ito K, Hofmann S, Matrix vesicles: role in bone mineralization and potential use as therapeutics. Pharmaceuticals (Basel). (2021) 14:289. doi: 10.3390/ph14040289 33805145 PMC8064082

[78] Akkus A, Karasik D, Roperto R. Correlation between micro-hardness and mineral content in healthy human enamel. J Clin Exp Dent. (2017) 9:e569–73. doi: 10.4317/jced.53345 28469825 PMC5410680

[79] Baldassarri M, Margolis HC, Beniash E. Compositional determinants of mechanical properties of enamel. J Dent Res. (2008) 87:645–9. doi: 10.1177/154405910808700711 18573984 PMC2658820

[80] Foster BL, Nociti FH Jr, Somerman MJ. The rachitic tooth. Endocr Rev. (2014) 35:1–34. doi: 10.1210/er.2013-1009 23939820 PMC3895863

[81] Ribeiro TR, Costa FW, Soares EC, Williams JR Jr, Fonteles CS. Enamel and dentin mineralization in familial hypophosphatemic rickets: a micro-CT study. Dentomaxillofac Radiol. (2015) 44:20140347. doi: 10.1259/dmfr.20140347 PMC4628496 25651274

[82] Opsahl Vital S, Gaucher C, Bardet C, Rowe PS, George A, Linglart A, Tooth dentin defects reflect genetic disorders affecting bone mineralization. Bone. (2012) 50: 989–97. doi: 10.1016/j.bone.2012.01.010 22296718 PMC3345892

[83] Socorro M, Shinde A, Yamazaki H, Khalid S, Monier D, Beniash E, Trps1 transcription factor represses phosphate-induced expression of SerpinB2 in osteogenic cells. Bone. (2020) 141: 115673. doi: 10.1016/j.bone.2020.115673 33022456 PMC7680451

[84] Mobley CG, Kuzynski M, Zhang H, Jani P, Qin C, Napierala D. Dspp-independent effects of transgenic Trps1 overexpression on dentin formation. J Dent Res. (2015) 94:1128–34. doi: 10.1177/0022034515586709 25999324 PMC4530388

[85] Owen C, Chen F, Flenniken AM, Osborne LR, Ichikawa S, Adamson SL, A novel Phex mutation in a new mouse model of hypophosphatemic rickets. J Cell Biochem. (2012) 113:2432–41. doi: 10.1002/jcb.24115 22573557

[86] Chang GT, Jhamai M, van Weerden WM, Jenster G, Brinkmann AO. The TRPS1 transcription factor: androgenic regulation in prostate cancer and high expression in breast cancer. Endocr Relat Cancer. (2004) 11:815–22. doi: 10.1677/erc.1.00853 15613454

[87] Vidal O, Lindberg MK, Hollberg K, Baylink DJ, Andersson G, Lubahn DB, Estrogen receptor specificity in the regulation of skeletal growth and maturation in male mice. Proc Natl Acad Sci U S A. (2000) 97: 5474–9. doi: 10.1073/pnas.97.10.5474 10805804 PMC25853

[88] Chen JQ, Litton J, Xiao L, Zhang HZ, Warneke CL, Wu Y, Quantitative immunohistochemical analysis and prognostic significance of TRPS-1, a new GATA transcription factor family member, in breast cancer. Horm Cancer. (2010) 1:21–33. doi: 10.1007/s12672-010-0008-8 21761348 PMC10358063

[89] Chang GT, van den Bemd GJ, Jhamai M, Brinkmann AO. Structure and function of GC79/TRPS1, a novel androgen-repressible apoptosis gene. Apoptosis. (2002) 7:13–21. doi: 10.1023/A:1013504710343 11773701

[90] van den Bemd GJ, Jhamai M, Brinkmann AO, Chang GT. The atypical GATA protein TRPS1 represses androgen-induced prostate-specific antigen expression in LNCaP prostate cancer cells. Biochem Biophys Res Commun. (2003) 312:578–84. doi: 10.1016/j.bbrc.2003.10.154 14680804

[91] Serandour AA, Mohammed H, Miremadi A, Mulder KW, Carroll JS. TRPS1 regulates oestrogen receptor binding and histone acetylation at enhancers. Oncogene. (2018) 37:5281–91. doi: 10.1038/s41388-018-0312-2 29895970 PMC6169732

[92] Wang L, Lu W, Zhang L, Huang Y, Scheib R, Liu X, Trps1 differentially modulates the bone mineral density between male and female mice and its polymorphism associates with BMD differently between women and men. PloS ONE. (2014) 9:e84485. doi: 10.1371/journal.pone.0084485 24416236 PMC3885592

[93] Gaikwad SS, Gheware A, Kamatagi L, Pasumarthy S, Pawar V, Fatangare M. Dental caries and its relationship to malocclusion in permanent dentition among 12–15 year old school going children. J Int Oral Health. (2014) 6:27–30. PMC4229825 25395789

[94] Bernhardt O, Krey KF, Daboul A, Völzke H, Kindler S, Kocher T, New insights in the link between malocclusion and periodontal disease. J Clin Periodontol. (2019) 46:144–59. doi: 10.1111/jcpe.13062 30636328

