# Supplementary material for: Deficiency of Mineralization-Regulating Transcription Factor Trps1 Compromises Quality of Dental Tissues and Increases Susceptibility to Dental Caries
Source: Front Dent Med. Author manuscript; Available in PMC 2022 May 13. (PMC9106314; doi:10.3389/fdmed.2022.875987)
Supplement: Supplementary Materials [file NIHMS1804532-supplement-Supplementary_Materials.pdf]

Supplementary Material

1.1 Supplementary Figures

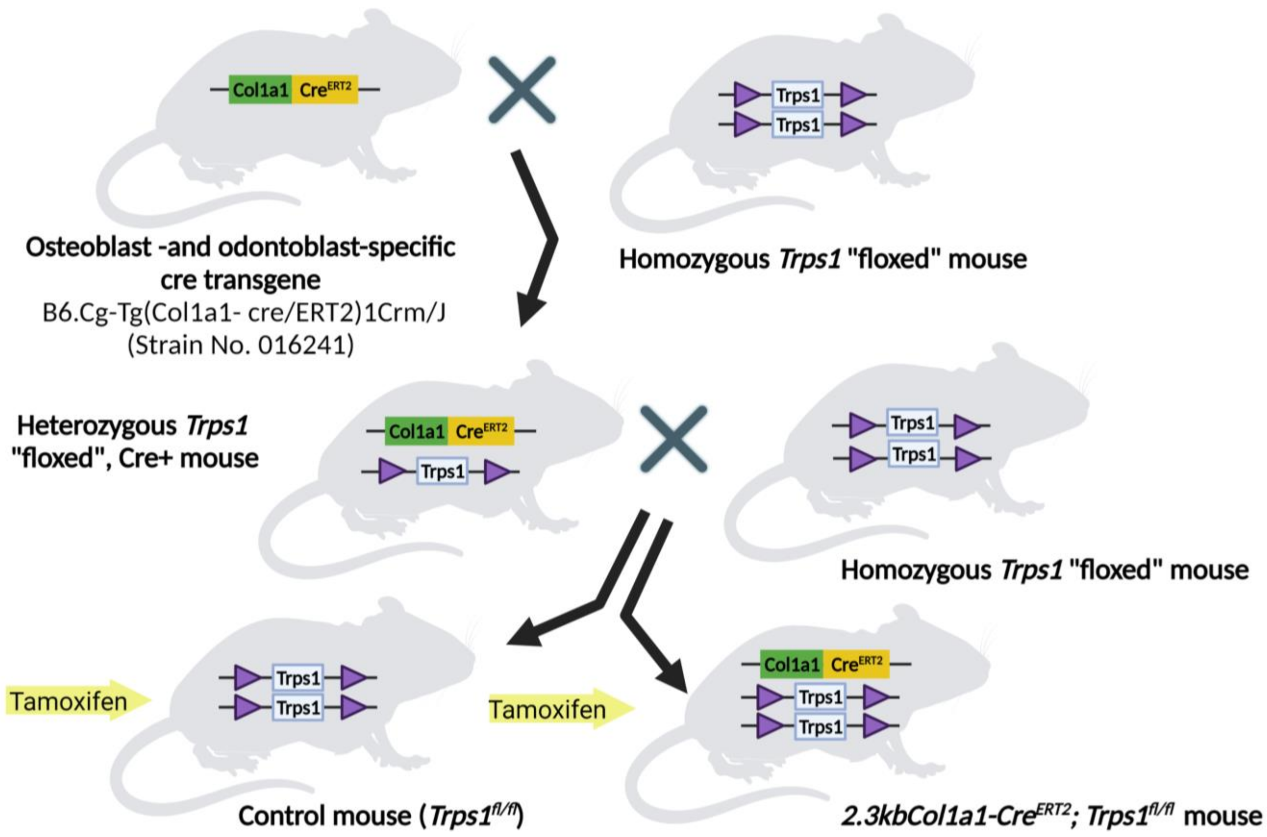

Supplementary Figure 1. Breeding scheme for generation of *2.3kbCol1a1-Cre*^{ERT2};*Trps1*$^{fl/fl}$ mice, to establish inducible odontoblast-specific *Trps1*-deficient mice. Male *2.3kbCol1a1-Cre*^{ERT2} mice were bred to homozygous *Trps1*$^{fl/fl}$ females (C57BL/6J background). Heterozygous progeny of the 1st generation was crossed with *Trps1*$^{fl/fl}$ females. The expansion pairs yield all necessary experimental mice (WT and *2.3kbCol1a1-Cre*^{ERT2};*Trps1*$^{fl/fl}$), which require tamoxifen administration for Cre activation. Purple triangles: LoxP sites. (Schematic modified and printed with permission from © The Jackson Laboratory).

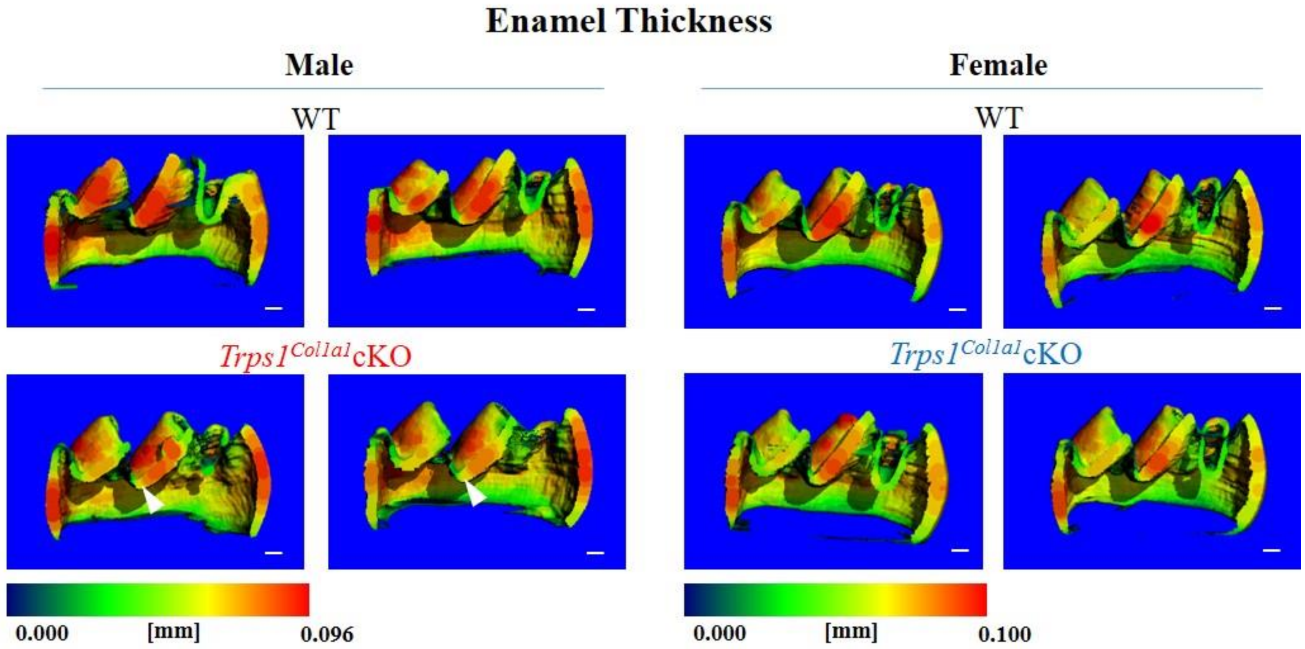

Supplementary Figure 2. µCT analyses of mandibular first molars. The enamel was pseudo-colored based on the distribution of tissue thickness. The corresponding tissue thickness color scale is shown below the images. Note the localized enamel mineralization defects in pits, especially in *Trps1*^{Col1a1}cKO male molars (white arrowheads). Differences were not as evident in *Trps1*^{Col1a1}cKO female mice when compared to WT. Scale bar= 100µm.

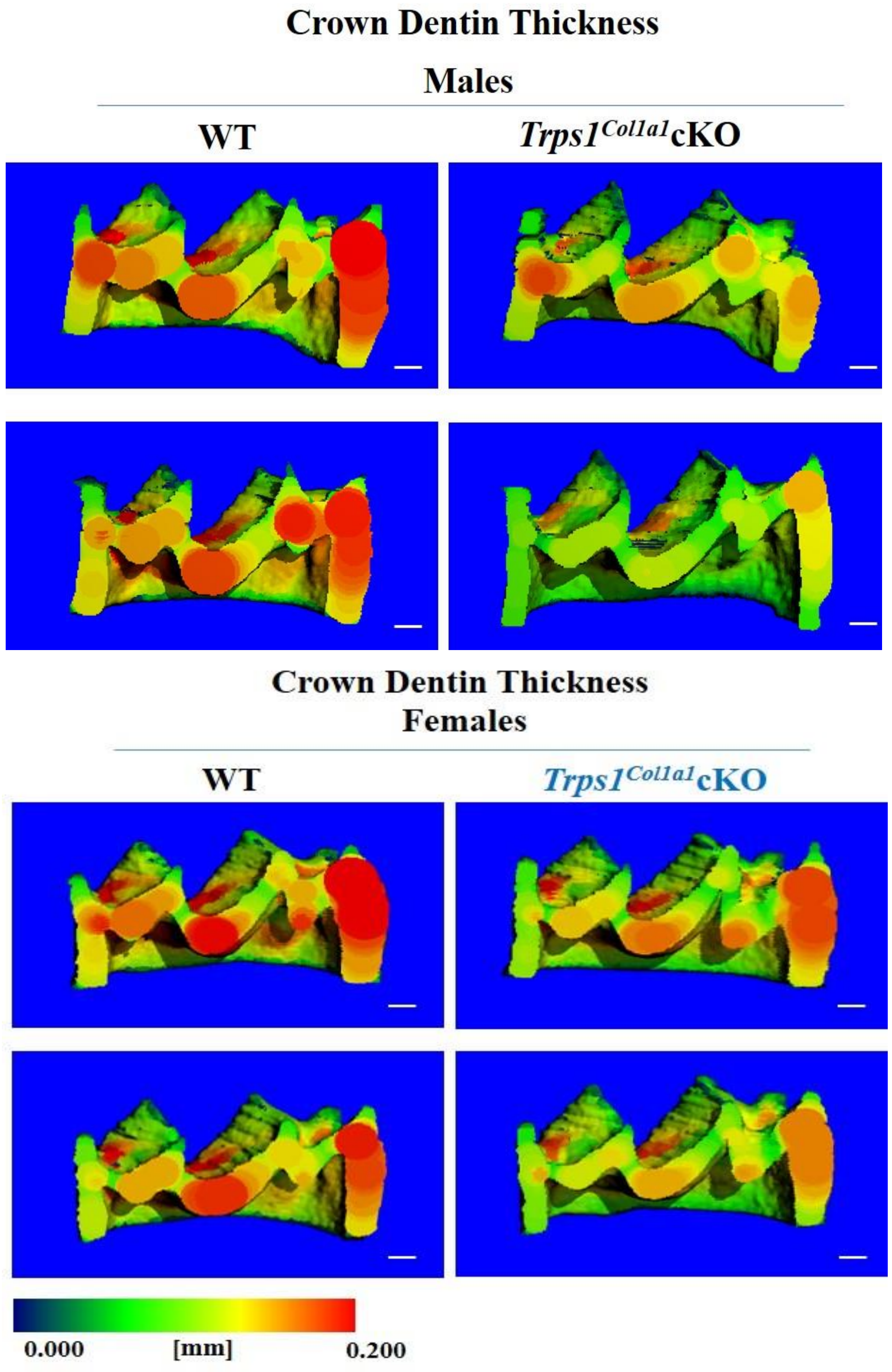

Supplementary Figure 3. µCT analyses of mandibular first molars. The dentin was pseudo-colored based on the distribution of tissue thickness. The corresponding tissue thickness color scale is shown below the images. Note overall thinner dentin in *Trps1*^{Col1a1}cKO molars versus WT. Scale bar= 100µm.

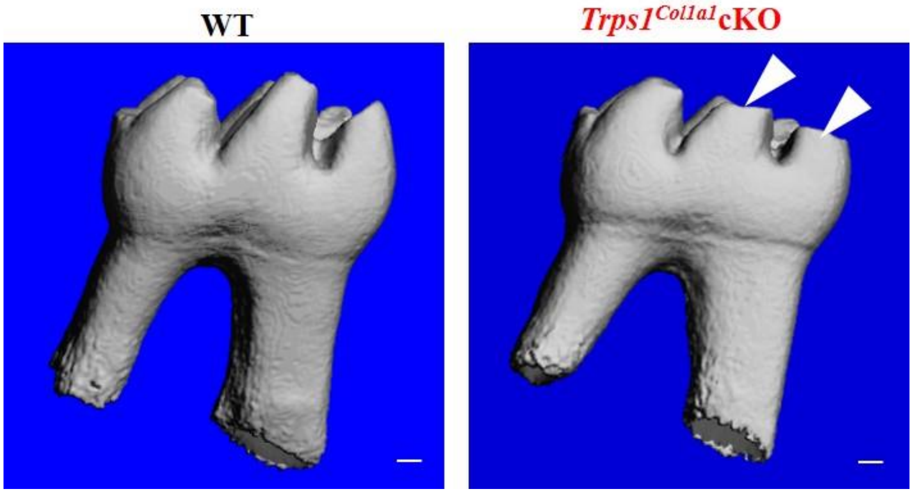

Supplementary Figure 4. µCT analyses of mandibular first molars. 3D-reconstruction images indicate areas with attrition in *Trps1*^{Col1a1}cKO male mice (white arrowheads). Scale bar= 100µm.
